# Discovery of novel RARα agonists using pharmacophore-based virtual screening, molecular docking, and molecular dynamics simulation studies

**DOI:** 10.1371/journal.pone.0289046

**Published:** 2023-08-24

**Authors:** Atefeh Ghorayshian, Mahshid Danesh, Tahereh Mostashari-Rad, Afshin fassihi

**Affiliations:** 1 Department of Cell and Molecular Biology, Faculty of Biological Science and Technology, University of Isfahan, Isfahan, Iran; 2 Functional Genomics & System Biology Group, Department of Bioinformatics, Biocenter, Am Hubland, University of Wuerzburg, Wuerzburg, Germany; 3 Department of Artificial Intelligence, Smart University of Medical Sciences, Tehran, Iran; 4 Department of Medicinal Chemistry, School of Pharmacy and Pharmaceutical Sciences, Isfahan University of Medical Sciences, Isfahan, Iran; The Islamia University of Bahawalpur Pakistan, PAKISTAN

## Abstract

Nuclear retinoic acid receptors (RARs) are ligand-dependent transcription factors involved in various biological processes, such as embryogenesis, cell proliferation, differentiation, reproduction, and apoptosis. These receptors are regulated by retinoids, i.e., retinoic acid (RA) and its analogs, as receptor agonists. RAR agonists are promising therapeutic agents for the treatment of serious dermatological disorders, including some malignant conditions. By inducing apoptosis, they are able to inhibit the proliferation of diverse cancer cell lines. Also, RAR agonists have recently been identified as therapeutic options for some neurodegenerative diseases. These features make retinoids very attractive molecules for medical purposes. Synthetic selective RAR agonists have several advantages over endogenous ones, but they suffer poor pharmacokinetic properties. These compounds are normally lipophilic acids with unfavorable drug-like features such as poor oral bioavailability. Recently, highly selective, potent, and less toxic RAR agonists with proper lipophilicity, thus, good oral bioavailability have been developed for some therapeutic applications. In the present study, ligand and structure-based virtual screening technique was exploited to introduce some novel RARα agonists. Pharmacokinetic assessment was also performed *in silico* to suggest those compounds which have optimized drug-like features. Finally, two compounds with the best *in silico* pharmacological features are proposed as lead molecules for future development of RARα agonists.

## 1. Introduction

Retinoic acid (RA), one of the primary active metabolites of vitamin A (retinol), is involved in regulating various biological processes such as immune responses, embryogenesis, homeostasis, cell proliferation, differentiation, apoptosis, and organogenesis [1, 2]. RA exists as at least six isomers (all-*trans*, all-*trans*-4-oxo, 9-*cis*, 9-*cis*-4-oxo, 13-*cis*, 13-*cis*-4-oxo) among them, all-*trans*-RA (ATRA) and 9-cis-RA (9cRA) are the most potent biologically active forms [3]. The conversion of ATRA into 9cRA and other isomers (as a reversible process) generates biological-active RA derivatives [4]. RAs or, more generally, retinoids exert their biological effects by binding as agonists to specific nuclear receptors called RA receptors (RARs) which exist in different types [5].

These three types of RARs are RAR-α, RAR-β, and RAR-γ belonging to the steroid/thyroid nuclear receptor superfamily [6, 7]. They share high sequence similarity and ligand-binding features [8]. RAR*α* is ubiquitously expressed and found in the majority of tissues. It has also been devolved in several diseases, most notably acute promyelocytic leukemia (APL) [9, 10]. RARβ can be involved in ontogenesis of the central nervous system (CNS) during the development and differentiation of epithelia in adults [11]. Since it regulates essential pathways associated with the tumor-suppressive effects of retinoids in various epithelial cells, RARβ signaling may play role as a potential tumor suppressor [12]. RARγ is involved in chondrogenesis, craniofacial morphogenesis, maintenance of squamous epithelia, and embryonic tail bud development [11].

Several natural and synthetic retinoid compounds have been identified as therapeutic agents for a number of diseases including cancer, dermatological disorders, and neurodegenerative diseases [13]. Some of them, such as the natural ATRA acting as pan-specific for all RAR isotypes have been very successful in the treatment of APL by inducing differentiation of leukemic cells. In addition, several synthetic analogs of RA as pan-specific activation for all RAR isotypes have emerged as promising anticancer drugs due to their antiproliferative and pro-apoptotic effects [14, 15]. However, undesired effects such as teratogenicity, bone toxicity, and serum lipid increment restricted further clinical application of these classes of therapeutic agents [16, 17]. Chemical modification of several functional groups of RA introduced some selective agonists with higher therapeutic efficacy and lower side effects compared with other pan-RAR agonists [18]. Along with these modifications, structural changes to improve the pharmacokinetic features of these lipophilic compounds resulted in highly selective, potent RAR agonists with suitable oral bioavailability [10].

Selective RARα agonists have been shown to inhibit cancer cell proliferation, induce apoptosis of mammary tumors, and inhibit LPS-induced B-lymphocyte proliferation [19, 20]. Selective RARα agonists cross the blood-brain barrier (BBB) and prevent neuronal cell death caused by amyloid-β (Aβ). These agonists are also able to inhibit Aβ production and control the Alzheimer’s disease (AD) progression [21]. Moreover, selective RARα agonists suppress the allospecific immune response, significantly prolong cardiac allograft survival, and can relieve lupus nephritis [22, 23].

RAR568 is a novel RARα-selective agonist with high oral bioavailability and a suitable pharmacokinetic profile. It has shown high oral bioavailability, over 80%, in both mice and dogs. RAR568 has no genotoxicity and cytotoxicity, demonstrating proper therapeutic potential [13, 24]. Recently, RAR568 treated regulatory T cells developed from patients with Crohn’s disease retrain the optimal phenotypic stability and suppressive capability compared with the standard culture conditions [25]. Although AM580 and AGN195183 also have appropriate selectivity for RARα over RARβ and RARγ, they are highly lipophilic (cLog P 6.3 and 7.2) with poor oral bioavailability [26, 27]. Moreover, AM580 has represented toxicity, and AGN195183 has been discontinued in Phase I of clinical trials for cancer treatment [13, 28].

Computer-aided drug design (CADD) approaches (i.e., structure-based drug design (SBDD) and ligand-based drug design (LBDD)) have recently been developed as beneficial tools in biochemical and pharmaceutical sciences. These approaches can significantly contribute to drug discovery, the development of lead compounds, and the reduction of experimental costs and time [29]. Structure-based and ligand-based pharmacophore model is a reliable tool for discovering new classes of compounds for a given therapeutic category [30]. Molecular docking and molecular dynamics simulation are also among the key tools widely applied to build, visualize and analyze molecular structures and their structure-activity relationship at the atomic level [31].

Different *in silico* methods have been used to identify selective ligands for the nuclear receptor (NR) superfamily members [32, 33]. Virtual screening (VS) approach has been used to recognize novel ligands for these receptors with a large variety of biological activities [34–44]. Most of the compounds obtained from this screening process had proper activities both *in vitro* and *in vivo* confirming the *in silico* predictions. Structure-activity relationship studies intended to optimize many of these compounds led to the identification of potent drug candidates [35, 43]. In a drug repositioning project, *in silico* screening of the previously FDA-approved drugs was used for introducing RAR ligands [36]. Concerning RARα, which is the main subject of the present study, Schapira *et al*. introduced two novel agonists with affinities at 50 nM, *in vitro*, in 2001. To achieve these molecules, they performed a VS procedure on a homology model of RARα [44]. In a different study, a homology model of RARα developed from the RARγ three-dimensional (3D) structure and estrogen receptor-α (ERα) was used to screen a library of 153,000 compounds using molecular docking simulations. Two novel RAR antagonists with low micromolar *in vitro* activities were suggested in this research. Although RARα was the target for molecular docking evaluations in this study, the *in vitro* affinities of the proposed compounds were higher for RARβ [45]. Park *et al*. presented a library of pocket conformational ensembles associated with thirteen different nuclear receptors (NRs), including RARα. They used the ensembles for VS of large compound databases to recognize their ligands. The validation methods indicated that the models were highly selective for the known active ligands [46]. In another report on VS experiment, a phenyl-thiazolyl-benzoic acid derivative (PTB) was introduced as a novel agonist of RAR and RXR (retinoid X receptor). This compound showed selectivity to RXRα and RARα, but not to PPARα, δ/β or γ (peroxisome proliferator-activated receptors). Further experiments elucidated that this compound acts as both a differentiation inducer and a proliferation inhibitor to leukemic cells [47]. (−)-Muqubilin A, a marine compound was identified as a PPARα/γ-RXRα agonist, RARα positive allosteric modulator, and validated further through *in vitro* and *in vivo* tests. Here again, VS of an in-house molecular library was the exploited method of study [48]. Li *et al*. performed a VS process, applying a ligand-based pharmacophore modeling based on a series of structurally diverse RARα agonists. They built two pharmacophore models considering the binding (KI pharmacophore model) and the efficacy (EC_50_ pharmacophore model) to RARα. *In vitro* tests for six obtained compounds displayed proper activities on leukemia cell lines and other tumors [49]. In most of these studies, the proper lipophilicity necessary for suitable oral bioavailability which is a challenging issue for most of the RAR ligands, was not considered for the proposed hit compounds.

As it was explained before, RARα is one of the most promising therapeutic targets, and RARα agonists are potential therapeutic agents against several vital diseases. Here we decided to use *in silico* approaches, including pharmacophore search, ADMET, molecular docking, and molecular dynamics simulation to find some potential novel RARα agonists based on the pharmacophore features and molecular shape of RARα selective ligands. Considering the inappropriacy of the agonists introduced in the previous studies, in terms of side effects, lacked suitable drug-like features, and resistance to cancer treatment, we planned to use the most efficient protocols to reach the most effective compounds.

## 2. Methods

In this research, a well-established protocol of virtual screening was applied to find some novel activators of retinoic acid receptor type α. In the first step of this procedure, a pharmacophore was constructed based on the interaction of RAR568, a selective activator of RARα with this receptor. In the second step, ten different databases were subjected to search for compounds with structural similarity to this pharmacophore. Pharmacokinetics and pharmacodynamics features of the obtained structures were evaluated in order to filter off more potent and drug-like compounds. Finally, compounds with proper *in silico* pharmacologic were subjected to more detailed investigation of ligand-receptor interactions.

### 2.1. Pharmacophore search

Pharmacophore search was carried out using Pharmit web server (https://pharmit.csb.pitt.edu/) [50]. Ten different databases were investigated using the Pharmer search method to identify pharmacophore hits by employing the complex structure of RARα_RAR568 [51]. Crystal structure of RARα was retrieved from RCSB Protein Data Bank (PDB; https://www.rcsb.org/, PDB ID: 3KMR at a resolution of 1.8 Å) [52]. Swiss-PDB viewer 4.1.0 (Spdbv) and Chimera 1.16 software (USCF, CA, USA, 2021) were used to visualized the 3D structures [53, 54]. Then, RAR568 was docked into the binding site of RARα using AutoDock 4.2 program [55], and the best-docked pose with a favorable interaction score was selected for pharmacophore search. Pharmit provides both pharmacophore-based and shape-based search types. This web server supports different pharmacophore features, i.e., hydrogen bond donors (HBD), hydrogen bond acceptors (HBA), hydrophobic centroids (HYP), aromatic rings (Aro), positive ions (PI), and negative ions (NI). They can be eliminated or remained unchanged for the pharmacophore search according to the operator’s decision. The search was set up to select only one orientation for each conformation of pharmacophore hits. Some more filters, such as molecular weight (MWT) ≤ 500 g/mol and LogP ≤ 5 were also applied. The root mean square deviation (RMSDs) were calculated based on the aligned structures of the reference and hit molecules. Finally, all databases were subjected to screening process to find molecules with the same structural features. The hit molecules were sorted in a descending value of pharmacophore RMSD, and the ones with an RMSD less than or equal to two angstroms (Å) (RMSD≤ 2) were selected.

### 2.2. Pharmacokinetics assessment

Values of physicochemical features determining the pharmacokinetic properties of a molecule, i.e., absorption, distribution, metabolism, excretion, and toxicity (ADMET) were investigated for each of the molecules taken from the screening, using SWISSADME (http://www.swissadme.ch/) and PKCSM (http://biosig.unimelb.edu.au/pkcsm/prediction) web servers [56, 57]. The pharmacokinetics parameters, such as physicochemical descriptors, lipophilicity, water-solubility, drug-likeness characteristics, carcinogenicity, organ toxicity, and cytotoxicity were estimated. These web servers use the two-dimensional (2D) structures of the molecules or their SMILES codes to provide predictions on the above-mentioned parameters. The results can be saved in CSV file format.

### 2.3. Pharmacodynamics evaluation using high throughput docking

Molecules having appropriate ADMET characteristics were docked into the RARα binding site by AutoDock vina (ADV) implemented in the PyRx Virtual Screening Tool (http://PyRx.sourceforge.net/downloads) [58]. The molecules in SDF format were imported in PyRx environment and were changed to pdbqt format using PyRx ligand preparation option. Lamarckian genetic algorithm (LGA) was applied as the local search algorithm [59]. The parameters for LGA were as follows: initial population of 150 randomly placed individuals, 2500000 energy evaluations, a maximum number of 27000 generations, mutation rate of 0.02, and a crossover rate of 0.80. After uploading the pdbqt format of RARα, the grid box was set in a way that include all the key residues of the binding site and a little more space around this area with the following dimensions in Å: center (x, y, z) = (-5.54, -7.20, -11.93), dimensions (x, y, z) = (26.82, 32.76, 31.15) with an exhaustiveness of 8. The dockings were run and the results were provided in a CSV file and docking parameter file for each molecule. After analyzing the obtained data, molecules with the best docking scores were considered for the next studies.

### 2.4. Structure preparation and minimization

The X-ray crystal structures of RARs (α, β, and γ) in complex with their selective agonists were downloaded from RCSB Protein Data Bank (https://www.rcsb.org/) [52]. The agonists were removed from the complexes by Accelrys Discovery Studio Visualizer 4.0 software (DS 4.0, Accelrys Software.Inc., San Diego, CA, USA, 2014) [60] and RARs were saved as pdb files for the molecular docking process. The ligand preparation process for the high throughput docking process with ADV was explained in the previous section. The structures of all 18 selected small molecules for the final docking process with AutoDock 4.2 were drawn using ChemDraw program (ChemDraw Ultra15.0, Cambridge soft, USA, 2015) [61]. Hydrogens were added via HyperChem8 package (HyperChem 8.0.3, 2007) through model build option along with energy minimization using Geometry Optimization commands (MM+ force field and PM3 semi-empirical calculations) [62].

### 2.5. Docking with AutoDock 4.2

Molecular dockings of the molecules selected in the previous step were carried out by AutoDock 4.2 software [55]. The pdb crystallographic structures of RARs (α, β, and γ) were in complex with their corresponding selective agonists. The selective agonists for each RARs were re-docked. The final RMSDs were under 2 Å, thus, acceptable. AutoDockTools (ADT) 1.5.6 package was utilized to prepare the docking input files. All hydrogens were added to RARα, and Kolman charges were calculated. Subsequently, each non-polar hydrogen was merged with its corresponding carbon atom. Followed by specifying the torsion tree, small molecules and RARα files were saved in PDBQT format for the next step. The grid box was adjusted to 60 × 60 × 60 Å points in xyz directions with 375 Å spacing set on the ligand-binding site. LGA was applied using the default values except for the number of GA runs, which was considered 150. Docking was performed on a rigid receptor, and small molecules were regarded as flexible. Later, binding modes of the complex structures in the final docking parameter files were analyzed by Discovery Studio program [60]. Eventually, the best binding modes were selected as initial structures for Molecular dynamics simulation.

### 2.6. Molecular dynamics simulations

Molecular dynamics (MD) simulations of RARα, RARα in complex with RAR568 and compounds that showed the best interactions with the receptor according to the molecular docking studies were performed by AMBER14 package using ff14SB and Generalized Amber (GAFF) force fields [63–65]. The AM1-BCC partial atomic charges were calculated for RAR568 and the selected compounds using Antechamber module (Amber tools 15) [66, 67]. The charge of each complex was neutralized by adding Na^+^ ions to the structures by xleap [63]. The complexes were solvated in an octahedral box of 10 Å layer of TIP3P water molecules. Minimization of the systems were carried out through 5000 steps of steepest-decent (SD) and 5000 steps of conjugate gradient (CG). In order to calculate non-bonded interactions by PME method, the cutoff distance was adjusted to 9 Å in the periodic boundary condition [68]. The system heating was gradually conducted from 0 to 300 K for 100 ps, employing Langevin thermostat by the NVT ensemble [69]. To restrict all bonds, including hydrogen atoms, the SHAKE algorithm was applied [70]. Moreover, the equilibration of system was performed for 100 ps in the NPT ensemble. Finally, MD simulations were run for 100 ns with the NPT ensemble. The coordinates were saved every 0.4 ps for further analysis.

### 2.7. Trajectory analysis

VMD 1.9.3 software (https://www.ks.uiuc.edu/Research/vmd) was used for examination and visualization of each MD simulation trajectory [71]. The trajectory analysis was performed using CPPTRAJ module from Amber Tools 15 for calculating the root mean square deviation (RMSD), root mean square fluctuation (RMSF), radius of gyration (Rg), potential energy (PE), hydrogen bonds (H-bonds), principal component analysis (PCA), and free energy landscape (FEL) [72]. XMGRACE 5.1.19 program (https://plasma-gate.weizmann.ac.il/Grace/) was employed for 2D plotting of graphs [73].

### 2.8. Molecular mechanics Poisson–Boltzmann surface area calculation

Molecular Mechanics–Poisson Boltzmann Surface Area (MM-PBSA) approach is applied to calculate the binding free energies (ΔGbind) of the complex structures [74]. The analysis of ΔG_bind_ was carried out using mmpbsa.py module in Amber Tools 15 [75]. 500 snapshots of each trajectory were extracted at equal intervals from the last 50 ns of simulation for calculating the final ΔGbind values.

## 3. Results & discussion

### 3.1. Pharmacophore search

**Fig 1:**
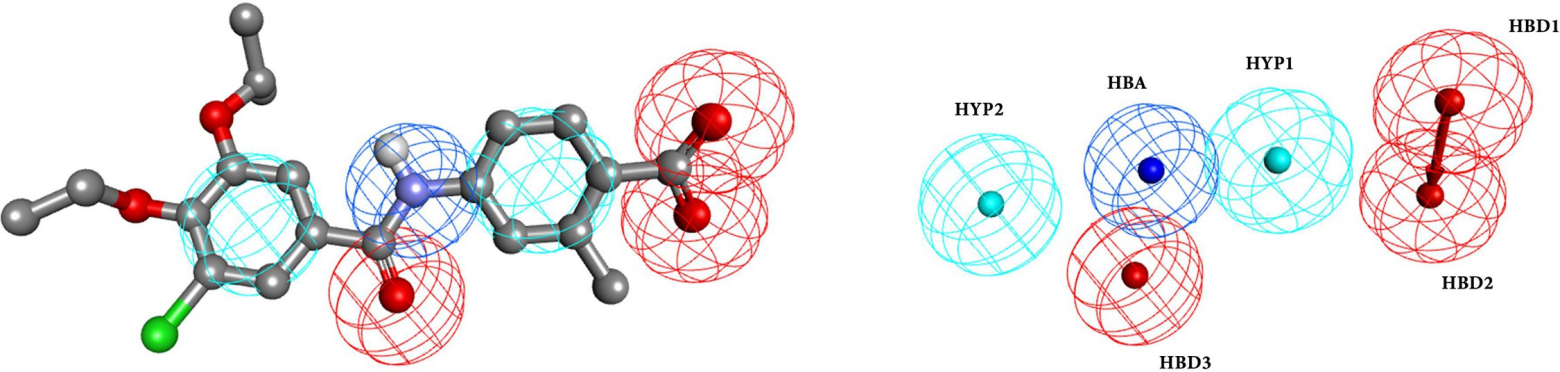
3D Pharmacophore model based on the pharmacophore features and molecular shape of RAR568 in the RARα binding site generated by Pharmit web server. Hydrogen bond donors (HBD) (blue sphere), hydrogen bond acceptors (HBA) (red spheres), and hydrophobic centroids (HYP) (cyan spheres).

A pharmacophore is an ensemble of molecular structure features, i.e., hydrogen bond donors (HBD), hydrogen bond acceptors (HBA), hydrophobic centroids (HYP), aromatic rings (Aro), positive ions (PI), and negative ions (NI). They are all crucial for molecular recognition of a given ligand by a specific biological macromolecule to trigger (or block) its biological response [76, 77]. A pharmacophore model may be applied to identify hit molecules with similar structural features against the binding site of the macromolecule target [78]. Pharmacophore search was carried out using Pharmit web server based on the pharmacophore features and molecular shape of RAR568. Moreover, the key interactions of RAR568 with amino acid residues at the binding site of RARα were investigated to identify pharmacophore hits via specific electronic and steric properties at various geometrical orientations responsible for the molecular activity [79, 80]. Pharmacophore features and/ or molecular shape provide an important insight to the functionalities which contribute in the molecular activity of hit molecules [81]. The essential characteristics of the pharmacophore model constructed in this research contained six pharmacophore points including one amide nitrogen atom to represent HBD feature, three negatively charge oxygen atoms to represent HBA features, and two aromatic rings to represent HYP features (Fig 1). Ten online chemical databases were search looking for structures similar to the pharmacophores proposed for RAR568. A series of hit molecules was collected from these databases (Table 1). Since RARα represents a linear “I” shaped binding site, conformationally extended compounds are accommodated better in the RARα active site [88]. In Sum, pharmacophore search resulted a collection of 12975 linear small molecules with MWT ≤ 500 g/mol, LogP ≤ 5, RMSD ≤ 2, and Energy Minimization -9≥, which were subjected to further analysis.

**Table 1:** List of online chemical databases used for pharmacophore search.

Entry	Database	Link.	Ref.
1	CHEMBL	https://www.ebi.ac.uk/chembl/	[82]
2	PubChem	http://pubchem.ncbi.nlm.nih.gov/	[83]
3	Zinc	http://zinc.docking.org/	[84]
4	MCULE	http://mcule.com/	[85]
5	MCULE-ULTIMATE	http://ultimate.mcule.com/	[85]
6	ChemDiv	http://www.chemdiv.com/	ChemDiv, Inc., San Diego, California
7	ChemSpace	http://chem-space.com/	[86]
8	MolPort	http://www.molport.com/	-
9	NCI Open Chemical Repository	http://dtp.cancer.gov/	[87]
10	LabNetwork	http://www.labnetwork.com/	**-**

### 3.2. ADMET assessment

Given that identification of new drug molecules is costly and time-consuming, several *in silico* approaches have emerged to predict and evaluate ADMET parameters for drugs and drug-like compounds prior to the synthesis [89]. The ADMET prediction was performed using SwissADME and pkCSM web servers to eliminate the weak small molecule drugs and identify the compounds with good potency.

Drug-like compounds have high similarities with the known drug molecules. They are preferably administered orally. Lipinski’s Rule of Five (ROF) is one of the most famous rules that indicates the features necessary for an oral drug [90]. It states that high oral absorption or permeation is more likely when no more than 5 HBD, 10 HBA, and 10 Rotatable bonds (RBN) exist in the molecular structure and the molecular weight and LogP are less than 500 g.mol^-1^ and 5, respectively [91]. Proper topological polar surface area as another measure of hydrogen bonding, thus, water solubility (TPSA<130) as well as a suitable lipophilicity Log P<5 were also considered.

Some more pharmacokinetics properties of agonist candidates such as gastrointestinal (GI) absorption, blood-brain barrier (BBB) permeability, the probability of being a substrate for efflux P-glycoprotein (Pgp), the possibility to inhibit two isoforms of cytochrome p450, CYP2D6 and CYP3A4, were also determined using SwissADME webserver. As discussed previously RARα agonists can cross the BBB and prevent Aβ production in AD [21]. P-glycoproteins are widely distributed throughout the body to limit cellular uptake and efflux xenobiotics and toxic substances from the cell [92]. CYP2D6 and CYP3A4 are two isoforms most important in metabolizing a wide range of drugs and xenobiotics. Thus, compounds with GI absorption, BBB permeability and no possibility to be a Pgp substrate or cytochrome P450 inhibitor were selected.

Ames toxicity, hepatotoxicity, and skin sensitization properties (related to toxicity) were identified using pkCSM webserver. The Ames test utilizes bacteria to investigate whether a given chemical can lead to mutations in the DNA of test organism. A positive test result indicates that the chemical is mutagenic and can act as a carcinogen, since cancer is often associated with mutation [93]. Hepatotoxicity is an uncommon but serious liver damage caused by exposure to drugs. Skin sensitization related to allergic contact dermatitis is a common health and occupational hazard resulting from an immunological response to chemical skin allergens [94].

Thus, molecules with no Ames Toxicity, no skin sensitization, and no probability of mutation were selected. According to the results, 938 compounds with the best ADMET features were considered for the following steps of the study.

### 3.3. High throughput docking using PyRx

PyRx Virtual Screening as one of the most promising *in silico* tools for analyzing the affinity of an extensive collection of compounds to a given receptor was applied in order to identify potent hit molecules among the 938 compounds obtained in the previous step. All of the 938 compounds were docked into the binding site of RARα to estimate their interaction scores. The estimated interaction scores of RARα agonist candidates were between -5.2 to -11.7 Kcal.mol^-1^ and 18 out of 938 compounds had the best interaction scores ≤ -10.7 and were selected for the detailed molecular interactions studies.

### 3.4. Molecular docking with AutoDock 4.2

**Fig 2:**
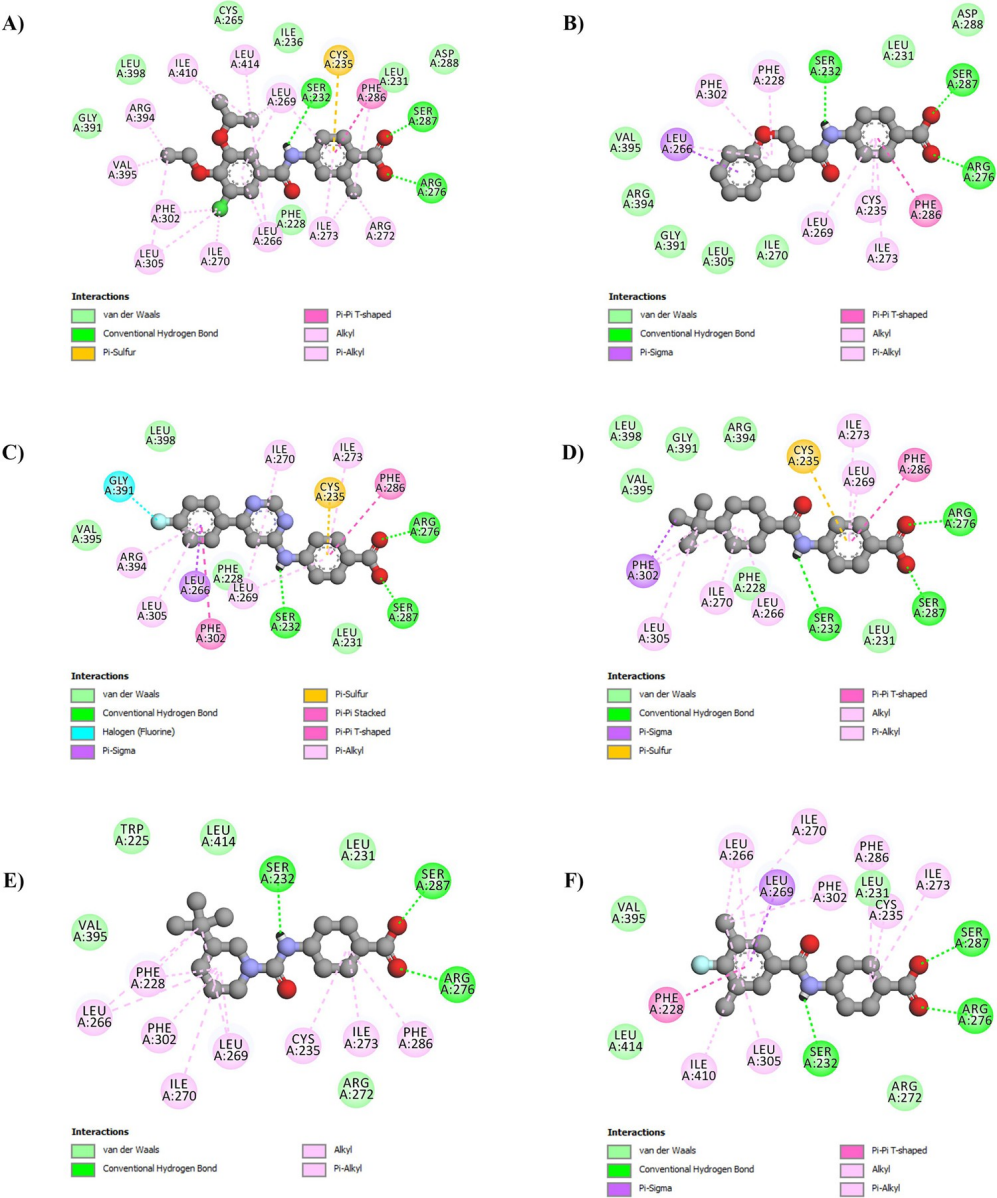
2D structure of the predicted interaction between RAR568 and the selected compounds with the RARα-substrate binding site. (A) RAR568, (B) Compound **1**, (C) Compound **2**, (D) Compound **4**, (E) Compound **8**, and (F) Compound **11** in interaction with RARα.

**Fig 3:**
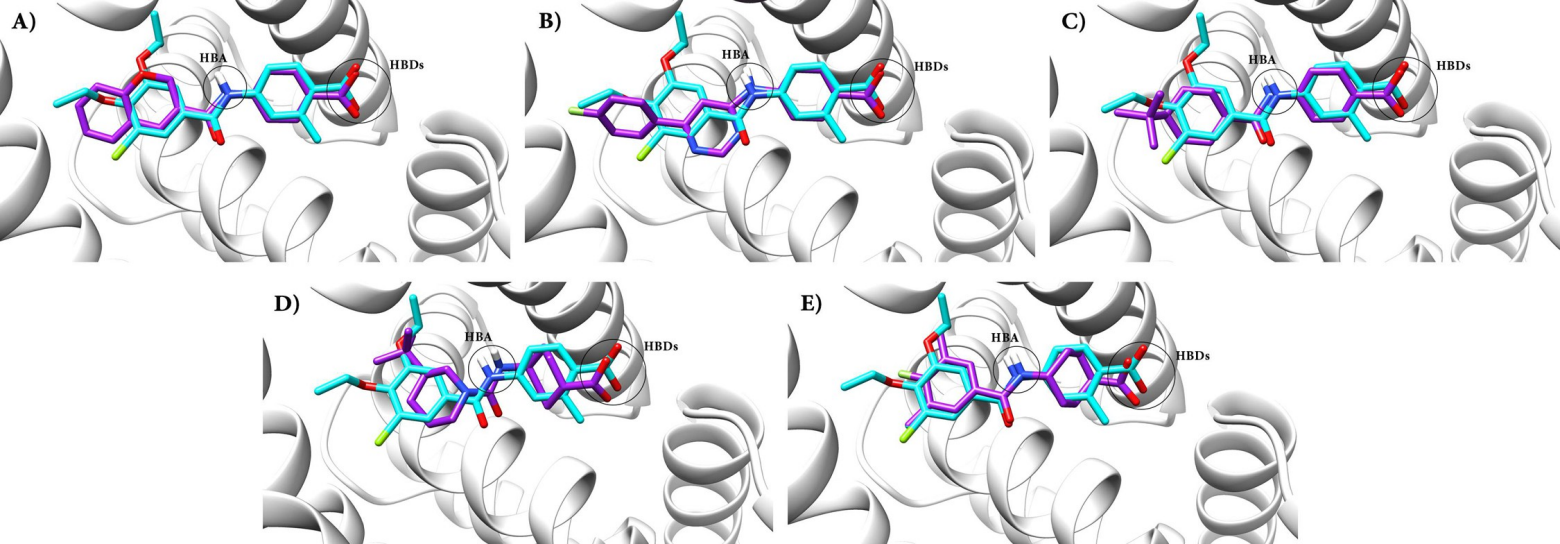
Superposition of the key pharmacophore features of RAR568 and the selected compounds with the RARα-substrate binding site. (A) Compound **1**, (B) Compound **2**, (C) Compound **4**, (D) Compound **8**, and (E) Compound **11** compared with RAR568 in interaction with RARα.

Molecular docking study was employed in order to assess the binding modes and determine the binding affinity between RARα and the selected molecules. The interactions between the key residues of RARα (Ser 232, Leu 266, Arg 276, Ser 287, and Arg 394) and the selected small molecules from the previous steps were compared with the ones with those between the RAR568 and RARα binding site. Initially, RAR568 was docked into the binding site of RARα leading to the results in agreement with the previous studies in this area [48, 95]. According to the structural evaluation of RARα-RAR568 complex, RAR568 had interaction with three key residues, including Ser232 (the most important key residue responsible for α-selectivity) [96], Arg276, and Ser287 by hydrogen bonds (Fig 2A). These interactions play the main biological role in the RARα agonists’ activity and selectivity [97, 98]. The interaction scores, as well as the intermolecular interactions of RAR568 and the selected molecules are tabulated in S2 Table in S1 File. As represented in this table, RAR568 and the selected molecules had almost similar interaction modes. The docking results indicated that among the studied molecules, compounds **1** and **2** compared with RAR568 had higher interaction scores (-10.99 and -10.73, respectively) and interacted with almost all key residues inside the RARα hydrophilic binding site. As can be observed in Fig 2B and 2C, compounds **1** and **2** formed three hydrogen bonds with the three key amino acid residues of the RARα binding site, including RARα-specific Ser 232, Arg 276, and Ser 287. Thus, these compounds were considered selective RARα agonists for the following steps. Furthermore, compounds **4**, **8**, and **11** with interaction scores of -10.56, -9.44, and -9.01, respectively, and interactions with the three key RARα-specific amino acids were also selected for the following steps of the studies (Fig 2D–2F). The key features of the pharmacophore interactions of RAR568 inside the RARα binding site were exactly represented by the selected compounds (Fig 3). HBA was formed between the nitrogen atom of the amide group of RAR568 and the five selected compounds with SER 232 inside the RARα active site. On other hand, HBDs were formed between the oxygen atoms of the RAR568 carboxyl group and all the selected compounds with SER287 and ARG276 residues.

In order to ensure the accuracy of the molecular docking technique as a method to distinguish the specific agonists for each subtype of RAR receptor, the specific agonist of RARγ, BMS184394, was docked into its active site [99]. The results showed that BMS184394 interacted with the key amino acid (methionine 272) in RARγ (S1A Fig) [100]. Furthermore, to investigate the RARα selectivity of the five studied compounds, they were docked inside the RARγ binding site. As illustrated in S1B–S1F Fig, none of them interacted with the key amino acid methionine 272, which is responsible for γ-selectivity, thus, can be regarded as specific RARα agonists. key residues for RARβ-selectivity have not yet been identified [101]. Based on sequence alignment, only three residues are different in the binding sites of α, β, and γ isoforms of RA receptor. Instead of Ser232 in RARα binding site, Ala225 and Ala234 in β and γ isoforms, respectively exist. Ile270 in α isoform has changed to Ile263 in β and Met272 in γ subtypes of RA receptor. Instead of Val395 in RARα binding site, Val388 in β and Ala 397 in γ isoforms have been detected [100, 102]. Therefore, given the small differences in the binding site of RARs and the lack of information for RARβ-selectivity, experimental investigations on RARs are recommended to obtain more structural information about the specific binding profiles, activities of newly identified agonists, and their specific functions.

### 3.5. Molecular dynamics simulations studies

Molecular dynamics simulations were performed to achieve a deeper comprehension of the structural dynamics and interactions of RARα in diverse structural combinations. Therefore, 100 ns MD simulations was carried out for RARα alone and for RARα in complex with RAR568 and each of the five compounds selected from the last molecular docking experiment. Root mean square deviation (RMSD), root mean square fluctuation (RMSF), radius of gyration (Rg), potential energy (Ep), hydrogen bonds (H-bonds), principal component analysis (PCA), and free energy landscape (FEL) were evaluated for each MD simulation.

**Fig 4:**
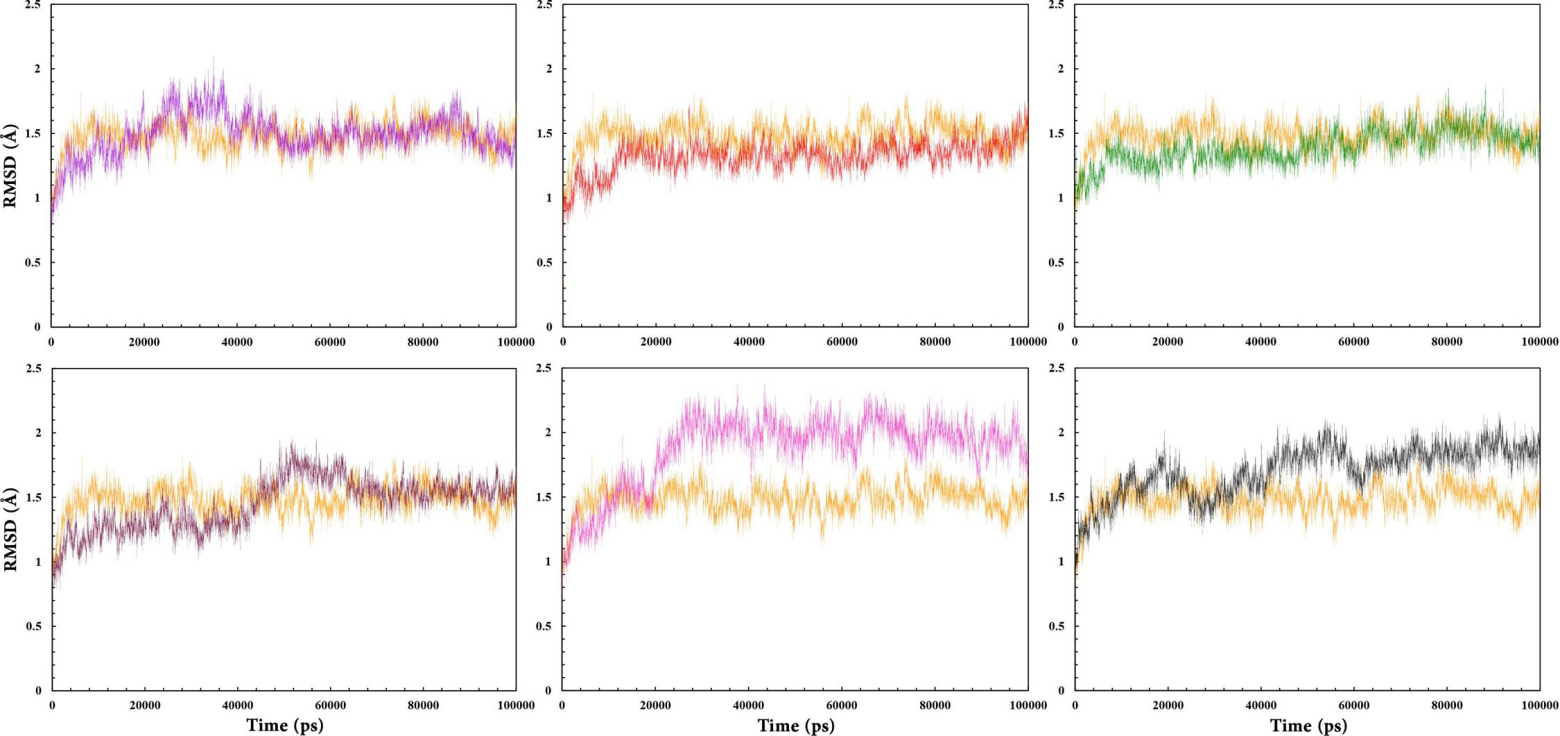
RMSD values of RAR568 and the five selected compounds in interaction with RARα as well as free RARα. RAR568 (Purple), Compound **1** (Red), Compound **2** (Green), Compound **4** (Magenta), Compound **8** (Pink), Compound **11** (Black), and free RARα (Orange).

In order to specify the stability of the complex structures, the RMSD was measured over the total simulation time [103]. The RMSD graph of all RARα complexes indicated that compounds **1**, **2**, **4**, and RAR568 had the lowest fluctuations in the range of 1.0–1.9 Å (Fig 4). In other words, they showed the highest level of stability throughout the MD simulations compared with the other compounds under the same simulation conditions. As illustrated in Fig 4, the RMSD values for compound **8** increased to 2.2 Å in the first 25 ns and remained stable through the rest of the simulation time in the range of 1.7–2.2 Å. The all-atom RMSD values for compound **11** also demonstrated small conformational changes in comparison with free RARα. The extent of fluctuations for this compound reduced after 60 ns in the range of 1.7–2.0 Å. These observations indicated that compounds **1**, **2**, and **4** with about 1.3 Å average RMSD form more stable RMSD profile complexes with RARα than the other compounds during the MD simulations.

**Fig 5:**
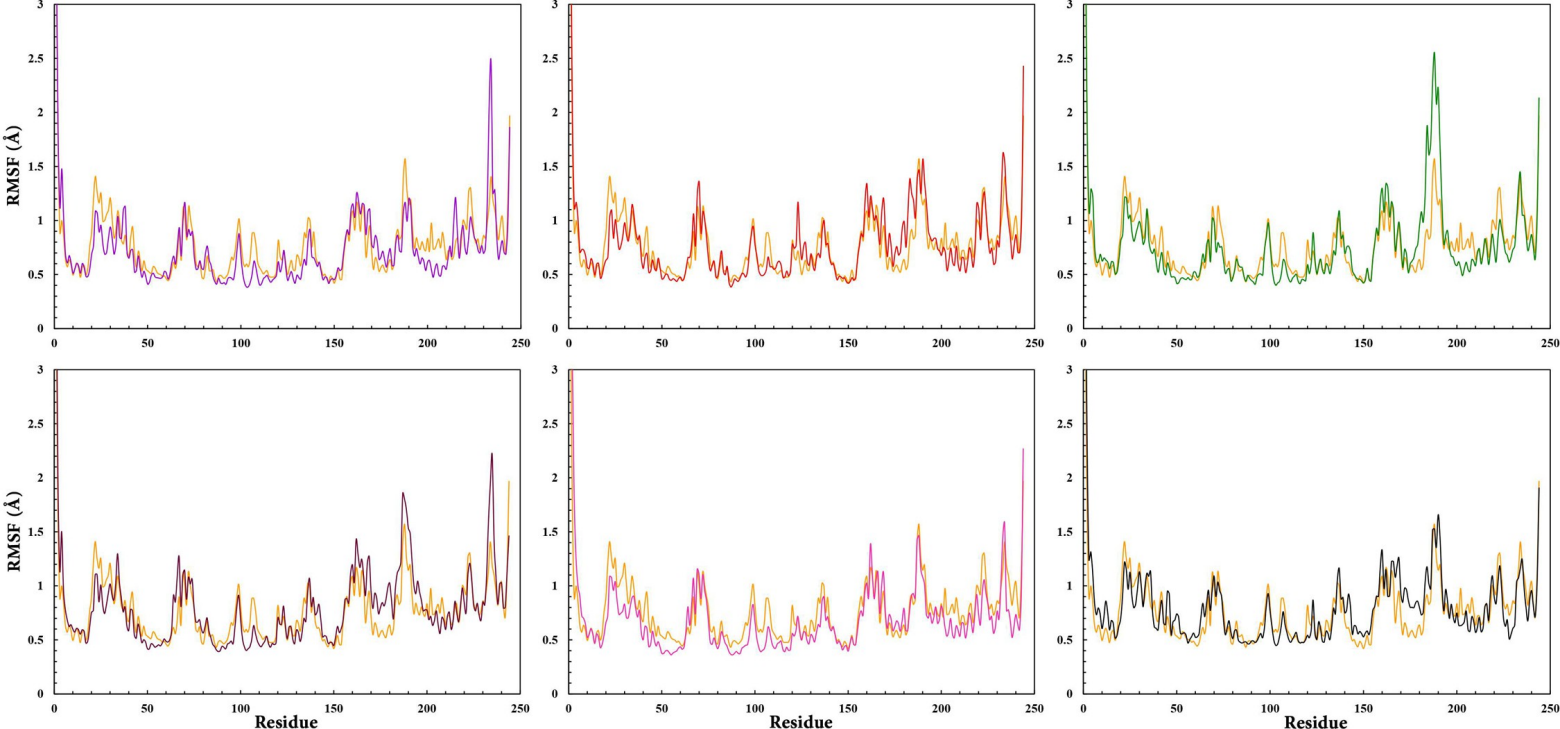
RMSF values of RAR568 and the five selected compounds in complex with RARα as well as free RARα. RAR568 (Purple), Compound **1** (Red), Compound **2** (Green), Compound **4** (Magenta), Compound **8** (Pink), Compound **11** (Black), and free RARα (Orange).

To characterize the regions in the complex structures exhibiting higher flexibility, the RMSF per residue values were employed [104]. The RMSF graphs of RARα models showed no remarkable difference in flexibility of the complexes, except in 180–190 and 230–235 nm (Fig 5). Higher values of RMSF displayed a higher rate of atomic mobility in backbone atoms (Cα) of RARα throughout the MD simulations. The key amino acid residues in the binding site (Ser232, Leu266, Arg276, Ser287, and Arg394) had the lowest value of RMSF in each MD simulation. The RMSF values of RAR568 and the selected compounds showed appropriate interactions and stable placement in the hydrophilic binding site of RARα throughout the MD simulations. The RMSF plot of free RARα also indicated a similar flexibility pattern during the MD simulation, suggesting that all compounds can act as potential agonists of RARα.

**Fig 6:**
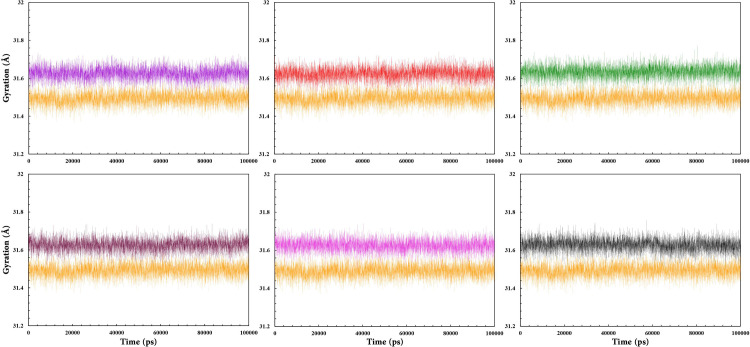
Rg values of RAR568 and the five selected compounds in complex with RARα as well as free RARα. RAR568 (Purple), Compound **1** (Red), Compound **2** (Green), Compound **4** (Magenta), Compound **8** (Pink), Compound **11** (Black), and free RARα (Orange).

The Rg changes provide information about the compactness level of the complex structures throughout the MD simulation [105]. According to Fig 6, the Rg graph for Cα atoms displayed an almost constant fluctuation for all complexes (31.65) over the total simulation time. The Rg plot of free RARα represented slightly less fluctuation (31.5) in comparison with the other complexes. This indicates that all compounds form relatively stable complexes on binding with RARα.

**Fig 7:**
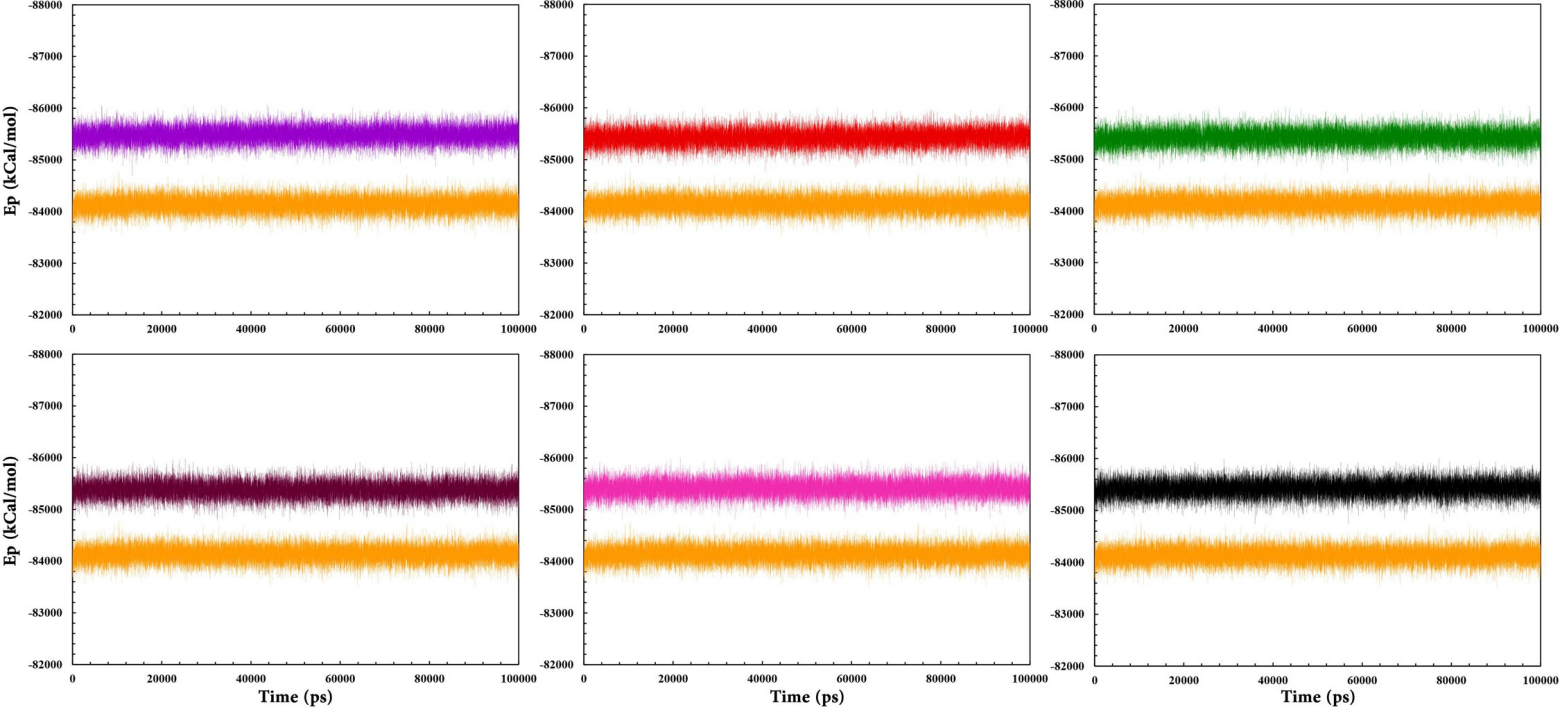
Ep values of RAR568 and the five selected compounds in complex with RARα as well as free RARα. RAR568 (Purple), Compound **1** (Red), Compound **2** (Green), Compound **4** (Magenta), Compound **8** (Pink), Compound **11** (Black), and free RARα (Orange).

Potential energy is the sum of bonded and non-bonded energies, demonstrating the stability of the complex structures [106]. The potential energy graph of all complexes showed fluctuations within the range of -85,000 to -86,000 (Fig 7). As can be observed from Fig 10, the potential energy of free RARα fluctuates in the range of -84,000. The average potential energy values for RARα, RAR568, and the other five selected compounds are tabulated in S3 Table in S1 File. According to this Table, all compounds indicated almost similar potential energy values and formed stable complexes with RARα.

**Fig 8:**
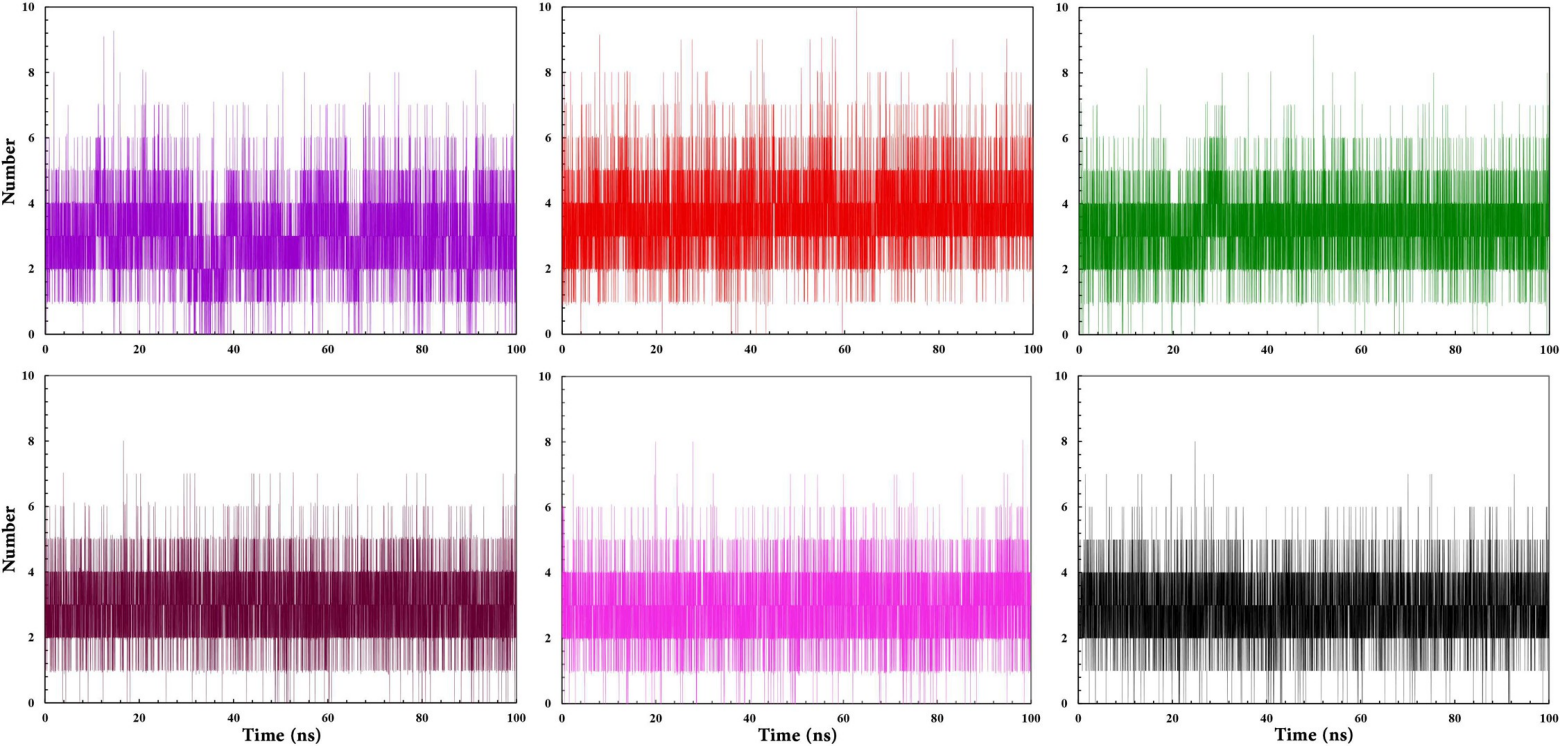
Hydrogen binding pattern of RAR568 and the five selected compounds in interaction with RARα. RAR568 (Purple), Compound 1 (Red), Compound 2 (Green), Compound 4 (Magenta), Compound 8 (Pink), and Compound 11 (Black).

Hydrogen bond interactions are significantly critical in stabilizing the complex structures throughout the MD simulation [107]. The H-bonds graph of all RARα complexes indicated that compounds **8** and **11** formed an average of 0–8 hydrogen bonds, compounds **2**, **4**, and RAR568 formed an average of 0–9 hydrogen bonds, while compound **1** formed an average of 0–10 hydrogen bonds (Fig 8). The overall result obtained from analyzing hydrogen bonds indicates that all compounds can form stable complexes with RARα. Compound **1** forms additional H-bonds with RARα, displaying its ability to form a more stable complex.

**Fig 9:**
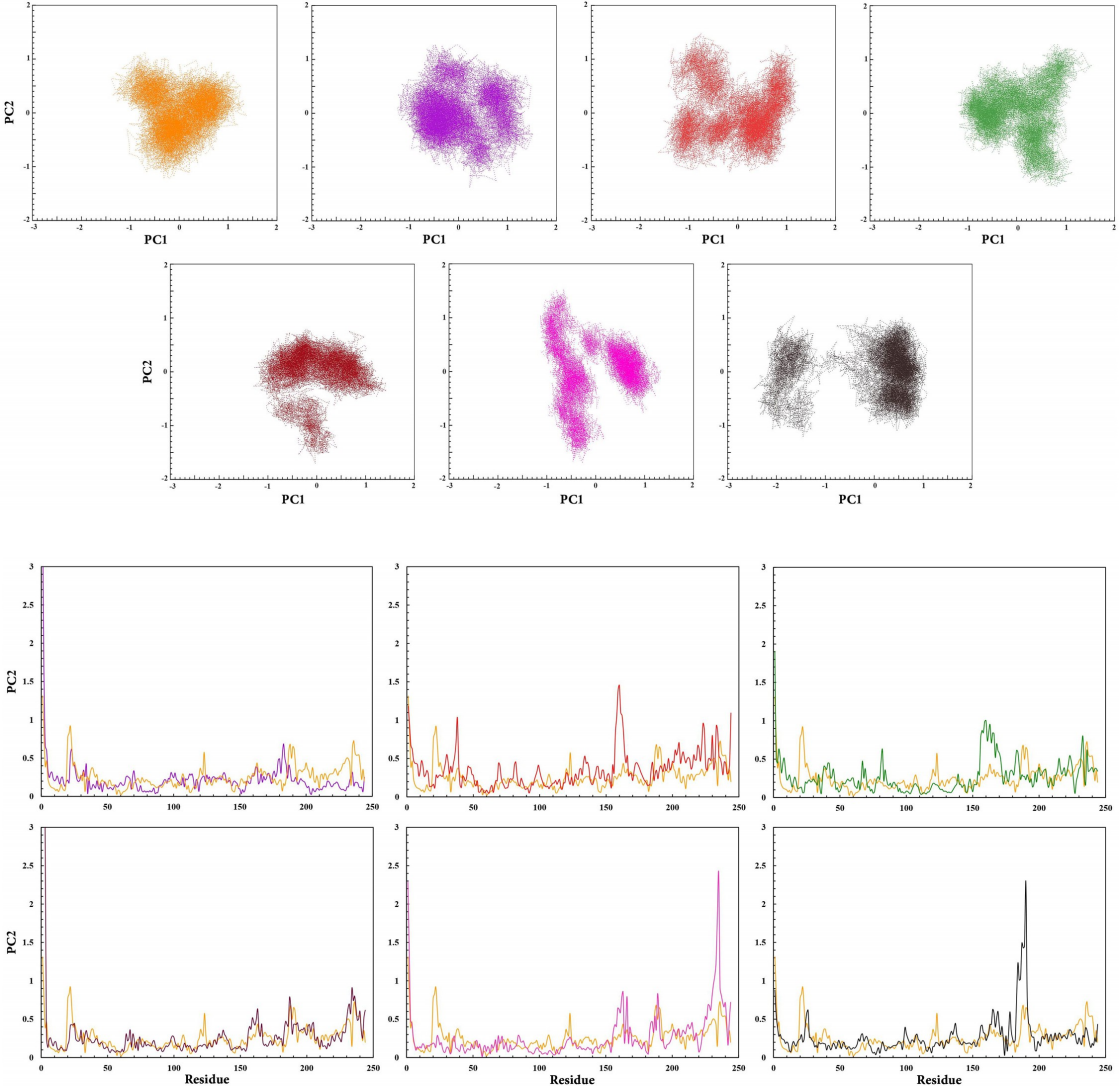
**A.** PCA scatters plot of RAR568 and the five selected compounds in interaction with RARα as well as free RARα on PCA parameters. Free RARα (Orange), RAR568 (Purple), Compound 1 (Red), Compound 2 (Green), Compound 4 (Magenta), Compound 8 (Pink), and Compound 11 (Black). **B.** RMSF values of RAR568 and the five selected compounds in interaction with RARα as well as free RARα in PC1 phase space. RAR568 (Purple), Compound **1** (Red), Compound **2** (Green), Compound **4** (Magenta), Compound **8** (Pink), Compound **11** (Black), and free RARα (Orange).

PCA indicated the collective motions of RARα as a result of RAR568 and agonist candidates binding over the entire phase space [108]. The scatter plot of RARα models represented different overall motions among the complex structures (Fig 9A). Evidently, compounds **2**, **4**, and RAR568 form more stable complexes with RARα than the other compounds. The motions were mainly due to the contribution between residue numbers 155–165 and 180–190 among all modes (Fig 9B). However, the key amino acid residues (Ser232, Leu266, Arg276, Ser287, and Arg394) had stable placement in the hydrophilic binding site of RARα as well as appropriate interactions with RAR568 and the selected compounds throughout the MD simulations. This indicates that all compounds can act as potential agonists of RARα.

**Fig 10:**
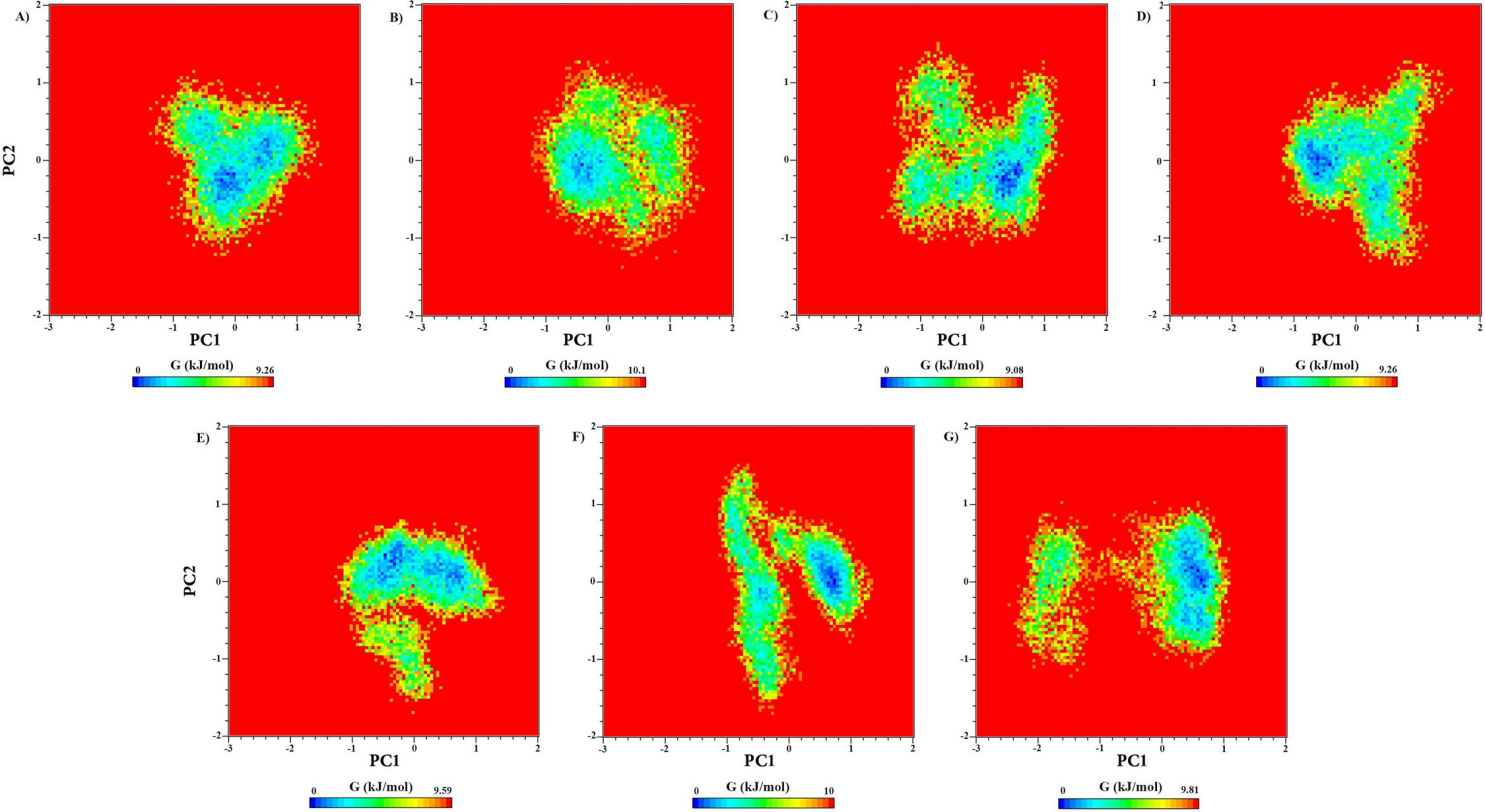
Free energy landscape of RAR568 and the five selected compounds in interaction with RARα as well as free RARα on PCA parameters. (A) free RARα, (B) RAR568, (C) Compound 1, (D) Compound 2, (E) Compound 4, (F) Compound 8, and (G) Compound 11.

Free energy landscape analysis was performed for Cα atoms of all complex structures in order to determine the thermodynamic stability [109]. The FEL graphs of RARα complexes indicated that compounds **1** and **2** had the lowest Gibbs free energy values between 0 to 9.08 and 0 to 9.26 kJ.mol^-1^, respectively (Fig 10). In other words, they displayed energetically more favorable molecular conformations in comparison with other complexes. The FEL graph of free RARα also showed the Gibbs free energy values between 0 to 9.26 kJ.mol^-1^. The overall results indicated that compounds **1** and **2** form more stable thermodynamically complexes with RARα throughout the MD simulations.

### 3.6. Analysis of binding free energies for RARα complex structures

**Fig 11:**
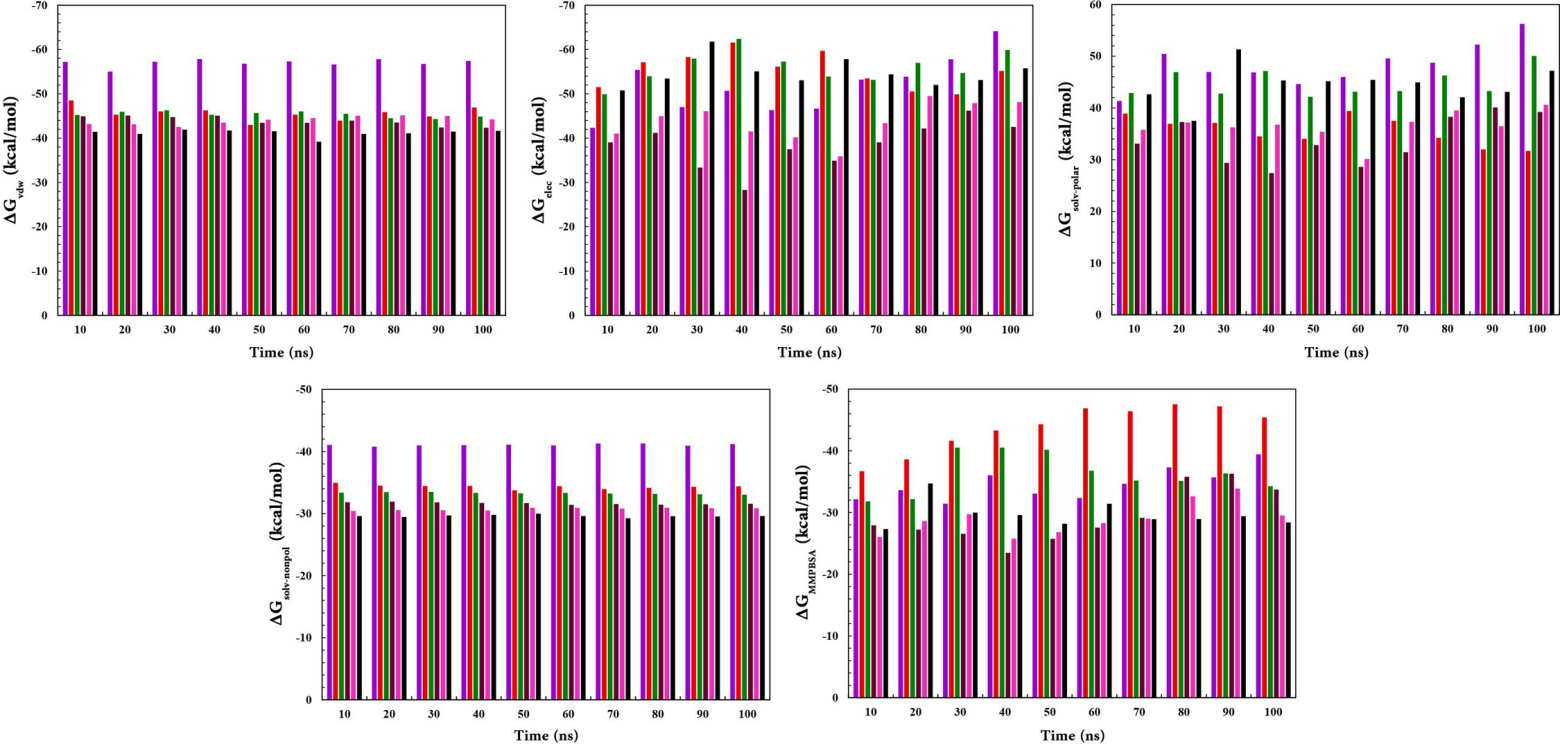
Free binding energies of RARα in complex with RAR568 and the five selected compounds with 10 ns interval during 100 ns using MM-PBSA approach. RAR568 (Purple), Compound 1 (Red), Compound 2 (Green), Compound 4 (Magenta), Compound 8 (Pink), and Compound 11 (Black).

MM-PBSA approach was carried out for calculating the binding energies of RARα in complex with RAR568 and the five selected compounds (Table 2). The results provided more information about the interaction mechanisms between RARα and agonist candidates (Fig 11). The best binding affinities were obtained for compounds **1** and **2** (compared with RAR568) with -44.6633 kcal.mol^-1^ and -35.5151 kcal.mol^-1^, respectively. This finding was in agreement with the docking results. According to MM-PBSA results, Van der Waals interactions had a critical role in binding affinities that were greater in compounds **1** and **2** compared with the other compounds. These findings are also corroborated by the literature [110, 111]. However, electrostatic interactions also played a decisive role in binding compounds to RARα. RAR568 and compound **4** showed the same binding affinities (-33 kcal.mol^-^1), and the lowest binding affinity was observed for compounds **11** and **8** with -29.6729 and -31.2386 kcal.mol^-1^, respectively.

**Table 2:** Total binding energies (kcal.mol^-1^) of RARα in complex with RAR568 and the five selected compounds retrieved by MM-PBSA approach.

	ΔG_vdw_	ΔG_elec_	ΔG_solv-polar_	ΔG_solv-nonpol_	ΔG_MMPBSA_
RAR568	-56.5179	-54.6833	52.5573	-41.2207	-33.6120
Compound **1**	-48.3491	-60.7011	39.4984	-35.1215	-44.6633
Compound **2**	-46.7502	-54.3880	44.7822	-33.7004	-35.5151
Compound **4**	-44.1089	-47.0058	36.7925	-31.9055	-33.0886
Compound **8**	-45.5478	-41.8187	39.8905	-31.1551	-31.2386
Compound **11**	-42.9726	-60.2224	51.3130	-29.9582	-29.6729

As far as we know, there is no report of MM-PBSA calculations for RARα. Thus, there are not any results for comparison with the results of the calculations in the present work. However, some studies have reported the MM-PBSA calculated energy for other members of the NR superfamily with their selective ligands, such as -20.19 kcal.mol^-1^ for RXRα and -13.13 kcal.mol^-1^ for RXRγ [112]. Since the structural similarity in the binding sites of RXRα and RXRγ is about 29 percent, no comparison with the MM-PBSA calculations results for RARα are possible. There are also some results of MM-PBSA calculations during 100 ns simulation for ERα (-23.77 kcal.mol^-1^), estrogen-related receptor-γ (ERRγ) (-26.69 kcal.mol^-1^), and PPARγ (-23.39 kcal.mol^-1^) [113]. However, since there was no *in vitro* confirmation for the aforementioned reported *in silico* results, it seemed unnecessary to regard them as references for comparison.

Therefore, the stability of the complex structures in this study can be discussed only in relation to RMSD, RMSF, Rg, PE, H-bonds, PCA, FEL and MM-PBSA resulted values. According to some reference articles that have investigated the ligand-binding domain of RARα, the main ligand-protein interactions were determined Van der Waals forces [110, 111]. This type of ligand-protein interaction was greater for compounds **1** and **2** compared with the other ones, indicating more stable protein-ligand complexes (Table 2).

Ultimately, based on the results, compounds **1** and **2** indicated more stable complexes and intermolecular interactions rather than the other compounds, free RARα, and the reference complex (RARα-RAR568).

## 4. Conclusion

Retinoids play crucial roles in regulating various biological processes due to their specific effects on cell proliferation, differentiation, and apoptosis. This makes them very attractive molecules for medical purposes. Retinoids exert their biological effects through binding to the nuclear retinoic acid receptors. Adverse effects restrict the further development and clinical application of these therapeutic agents. The ultimate goal of the present study was to introduce some potent and novel RARα-selective agonists with good drug-like features based on the RAR568 pharmacophore properties. Initially, pharmacophore search along with ADMET assessments and Virtual Screening were performed to generate a potential collection of RARα agonist candidates. Later, molecular docking and molecular dynamics simulation studies in combination with MM-PBSA approach were applied to evaluate the binding modes and binding affinities of the selected compounds with RARα, compared with RARα-RAR568 complex. Based on the findings, compounds **1** and **2** can be considered RARα agonists able to activate RARα and downstream effector mechanisms resulting in biological responses. The results provide indisputable *in silico* pieces of evidence about the affinity and selectivity of the compounds emerged from the screening protocol applied in this work. Experimental validation of the results obtained in this research will provide a deeper knowledge of the structural necessities of the compounds for selective attachment to the retinoic acid receptor α as an agonist. It might be one of the joint projects of our research group in the future.

## Supporting information

S1 Fig 2D structure of predicted interaction between BMS184394 and the other five selected compounds with the RARγ-substrate binding site. (A) BMS184394, (B) Compound **1**, (C) Compound **2**, (D) Compound **4**, (E) Compound **8**, and (F) Compound **11** in interaction with RARγ.
(TIF)

S1 Table ADMET prediction of RAR568 and 18 selected small molecules. (DOCX)

S1 File (ZIP)
