# Supplementary figures and images for: Discovery of novel RARα agonists using pharmacophore-based virtual screening, molecular docking, and molecular dynamics simulation studies
Source: PLoS One. 2023 Aug 24;18(8):e0289046. doi: 10.1371/journal.pone.0289046 (PMC10449137; doi:10.1371/journal.pone.0289046)

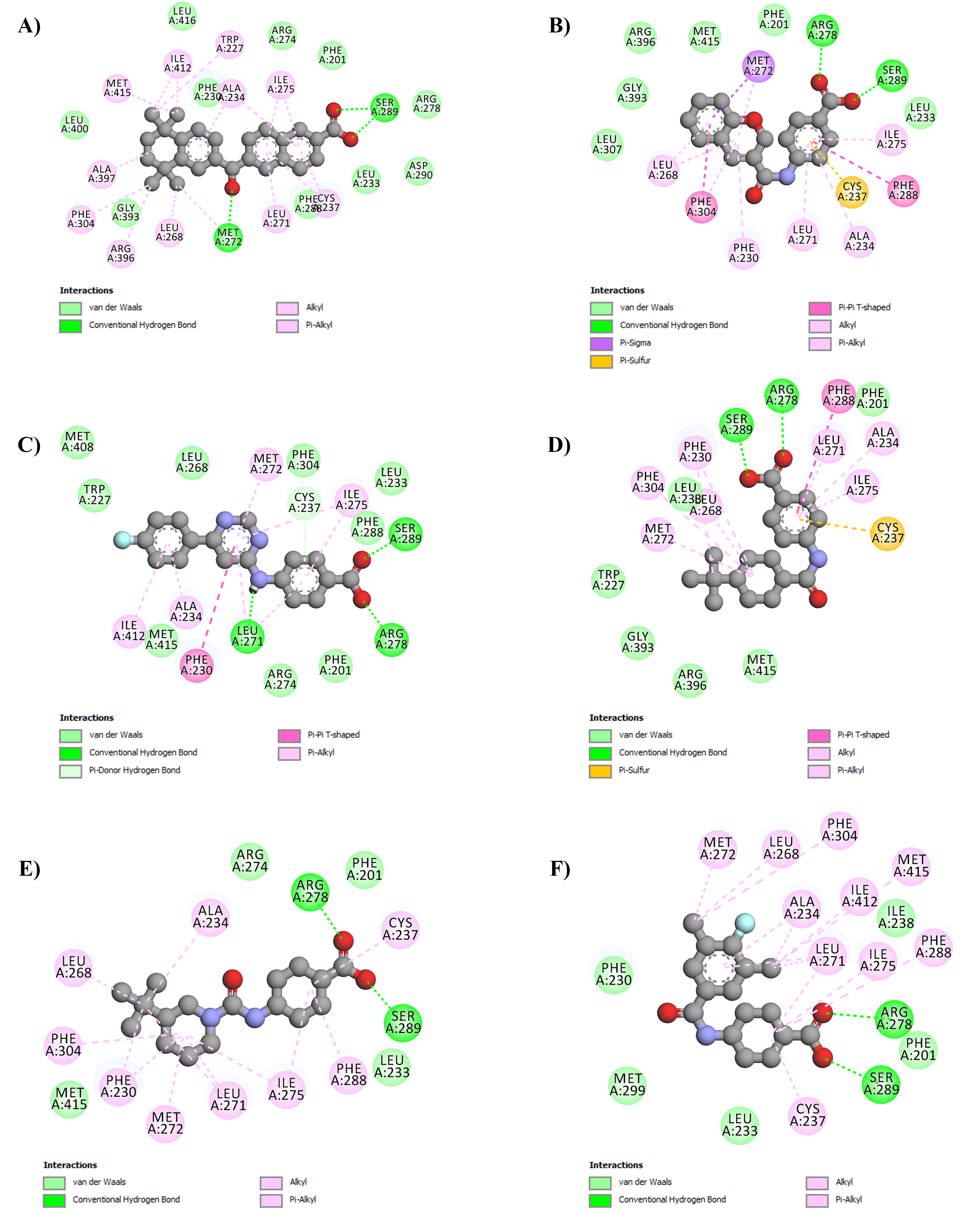

Supplement: S1 Fig — (A) BMS184394, (B) Compound 1, (C) Compound 2, (D) Compound 4, (E) Compound 8, and (F) Compound 11 in interaction with RARγ. (TIF) [file pone.0289046.s001.tif]

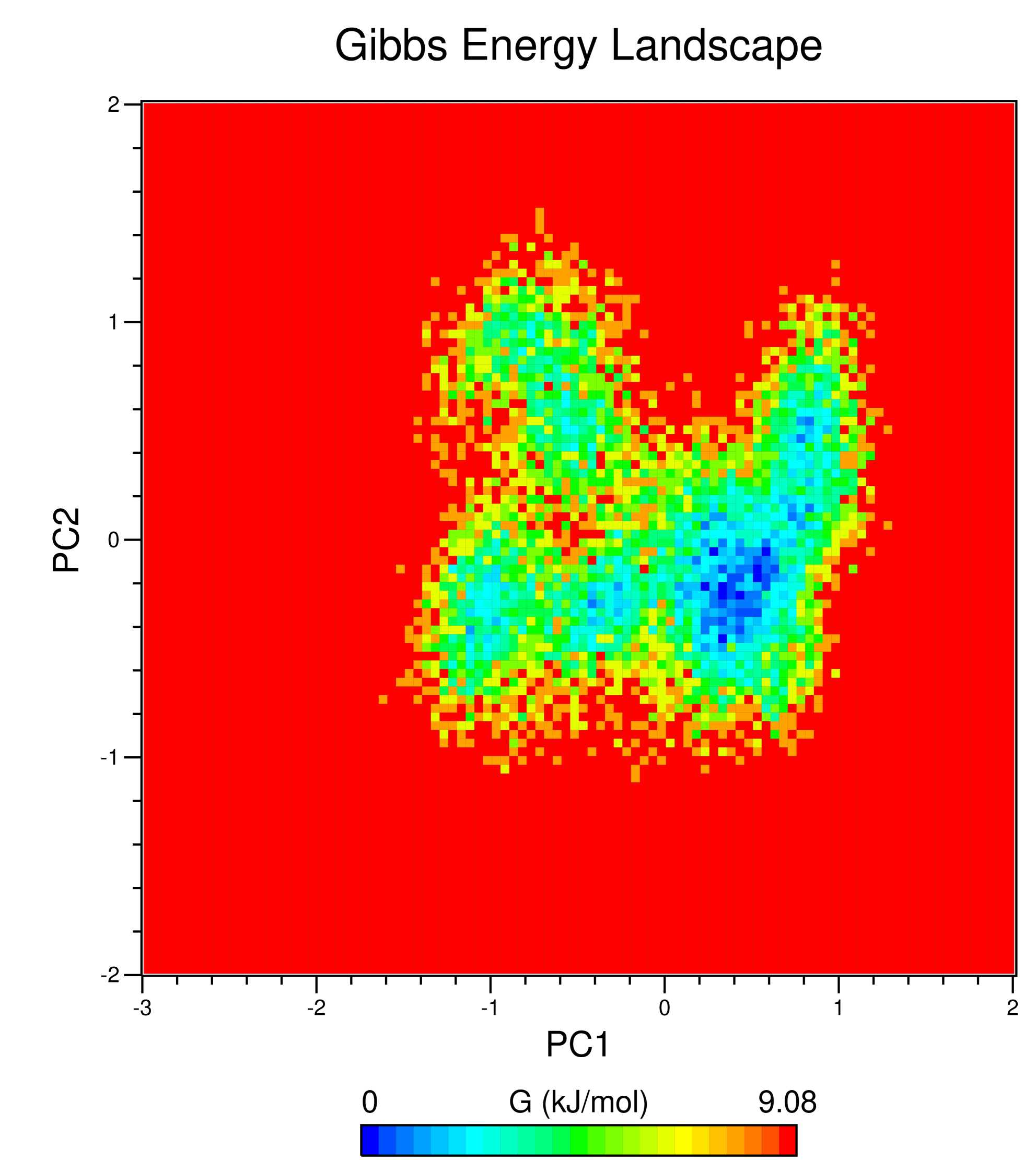

Supplement: S1 File — (ZIP) [file pone.0289046.s003.zip › Supplementary/Compound 1/MD/FEL/Gibbs energy.png]

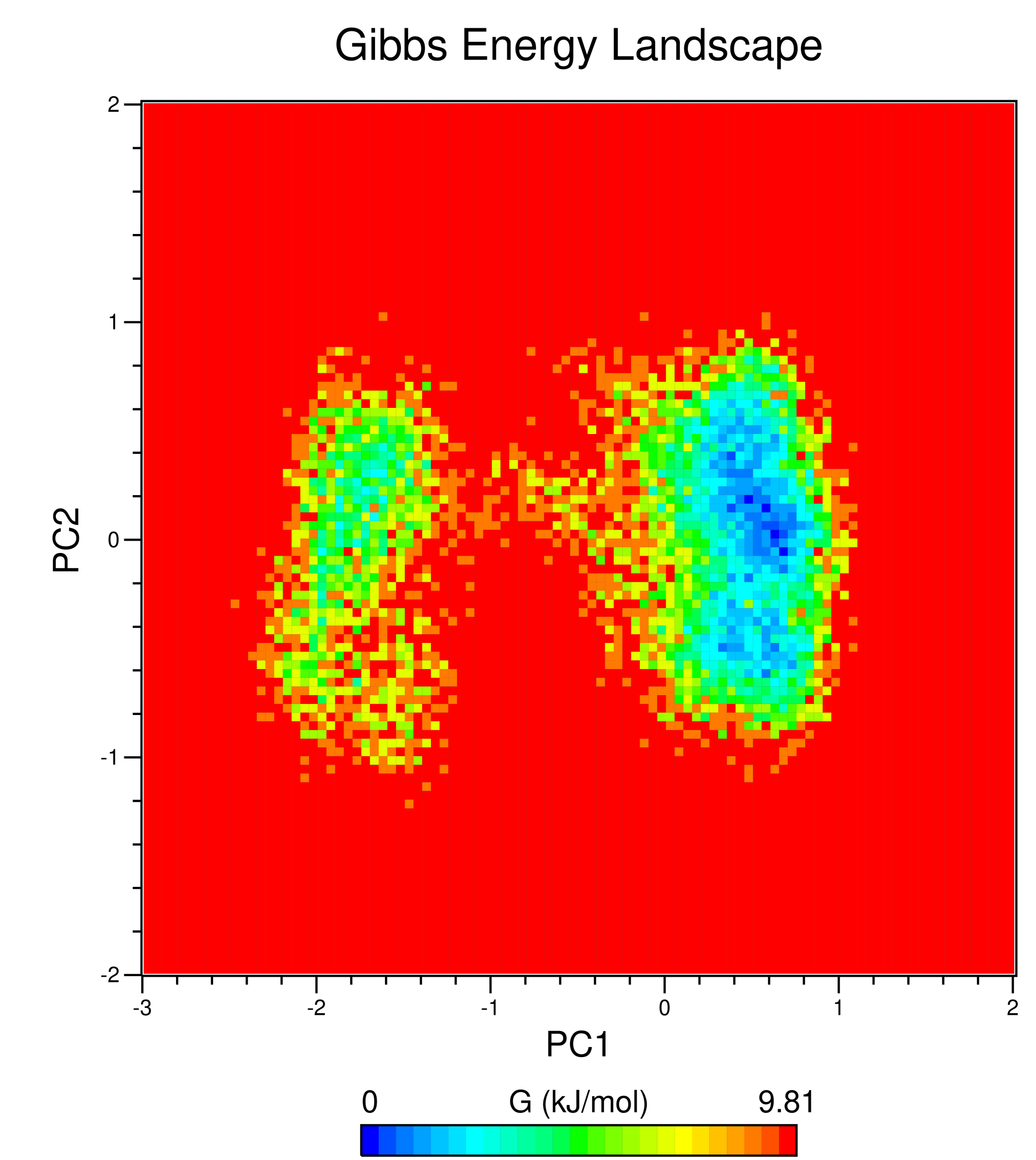

Supplement: S1 File — (ZIP) [file pone.0289046.s003.zip › Supplementary/Compound 11/MD/FEL/Gibbs energy.png]

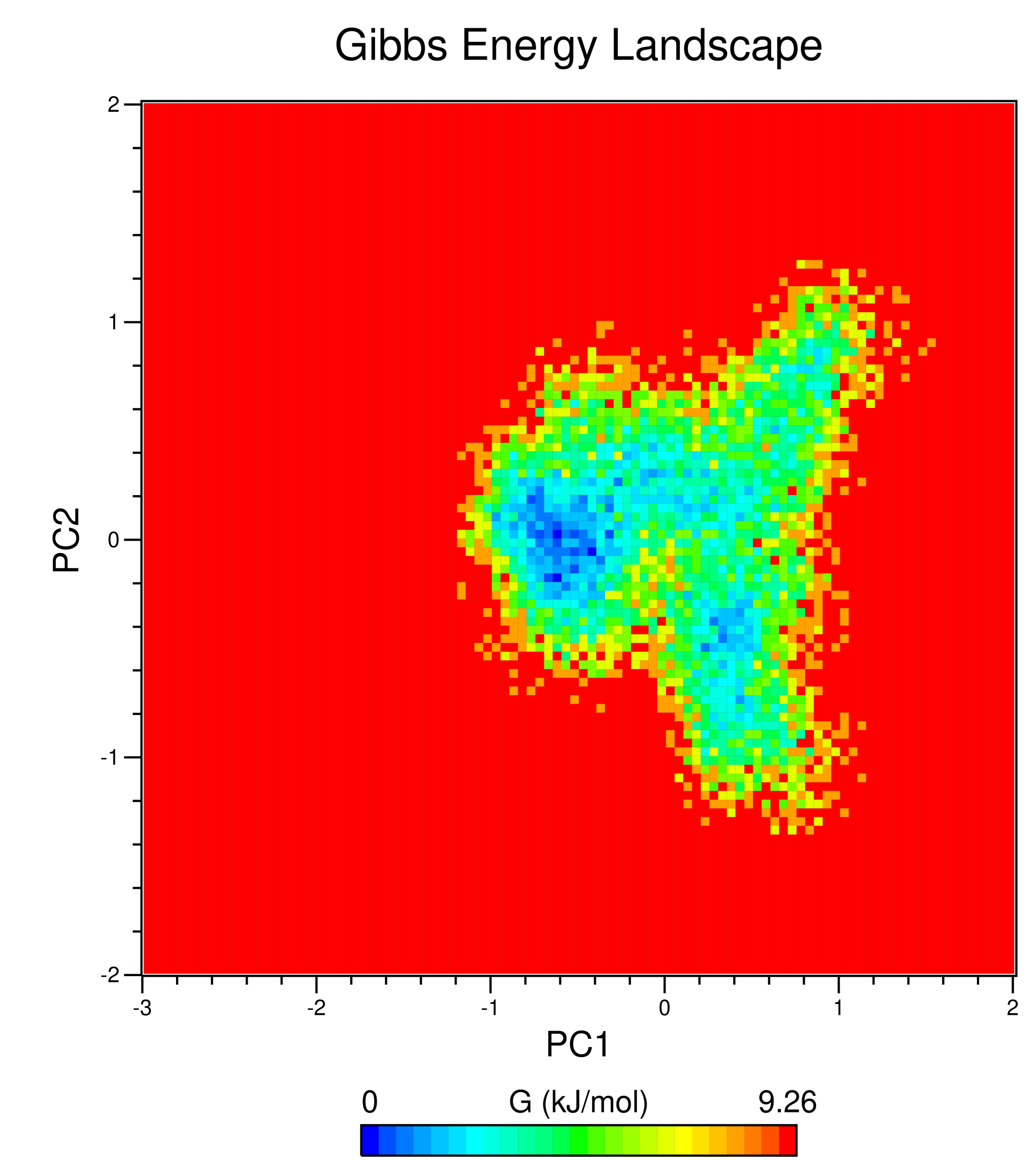

Supplement: S1 File — (ZIP) [file pone.0289046.s003.zip › Supplementary/Compound 2/MD/FEL/Gibbs energy.png]

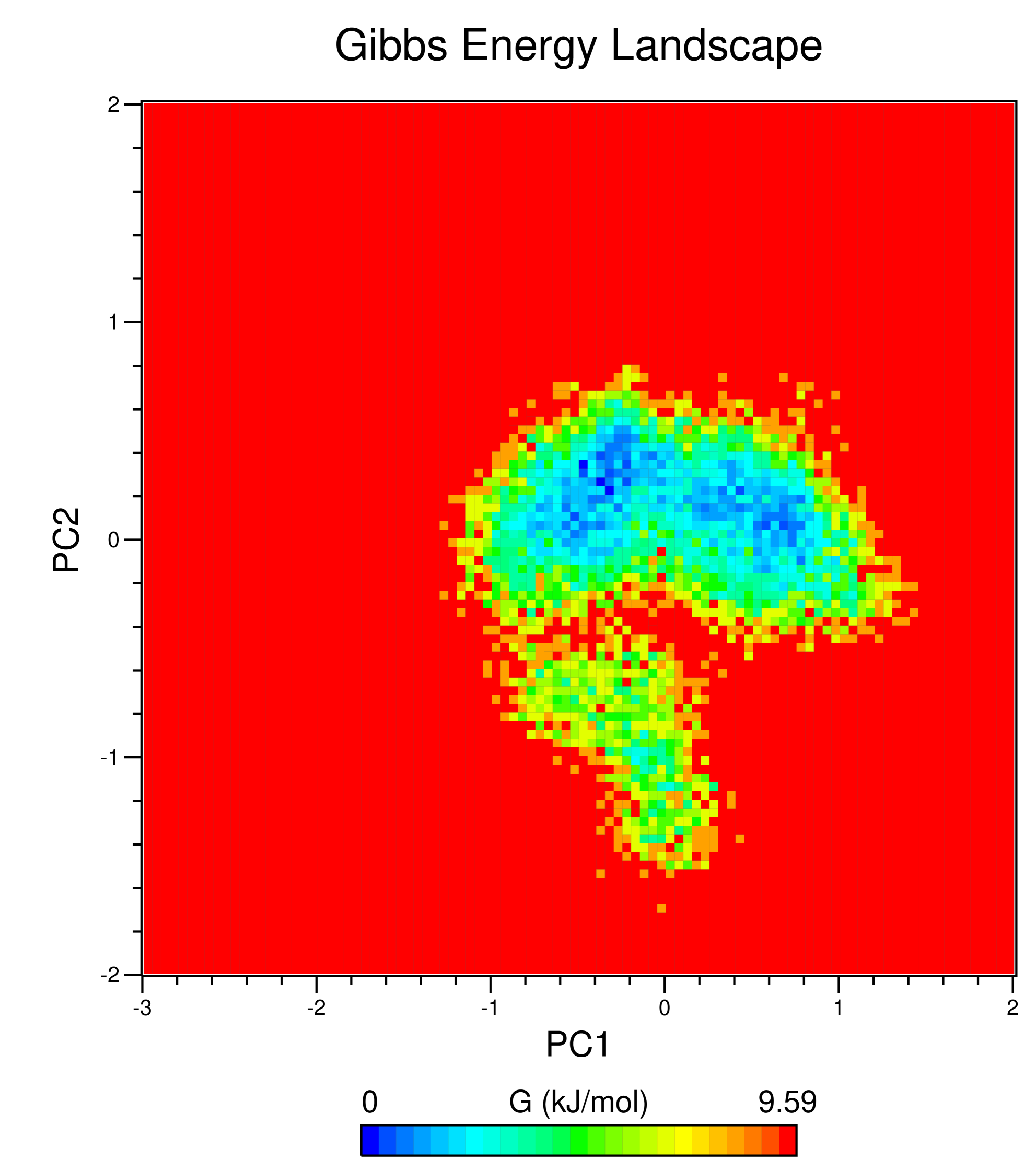

Supplement: S1 File — (ZIP) [file pone.0289046.s003.zip › Supplementary/Compound 4/MD/FEL/Gibbs energy.png]

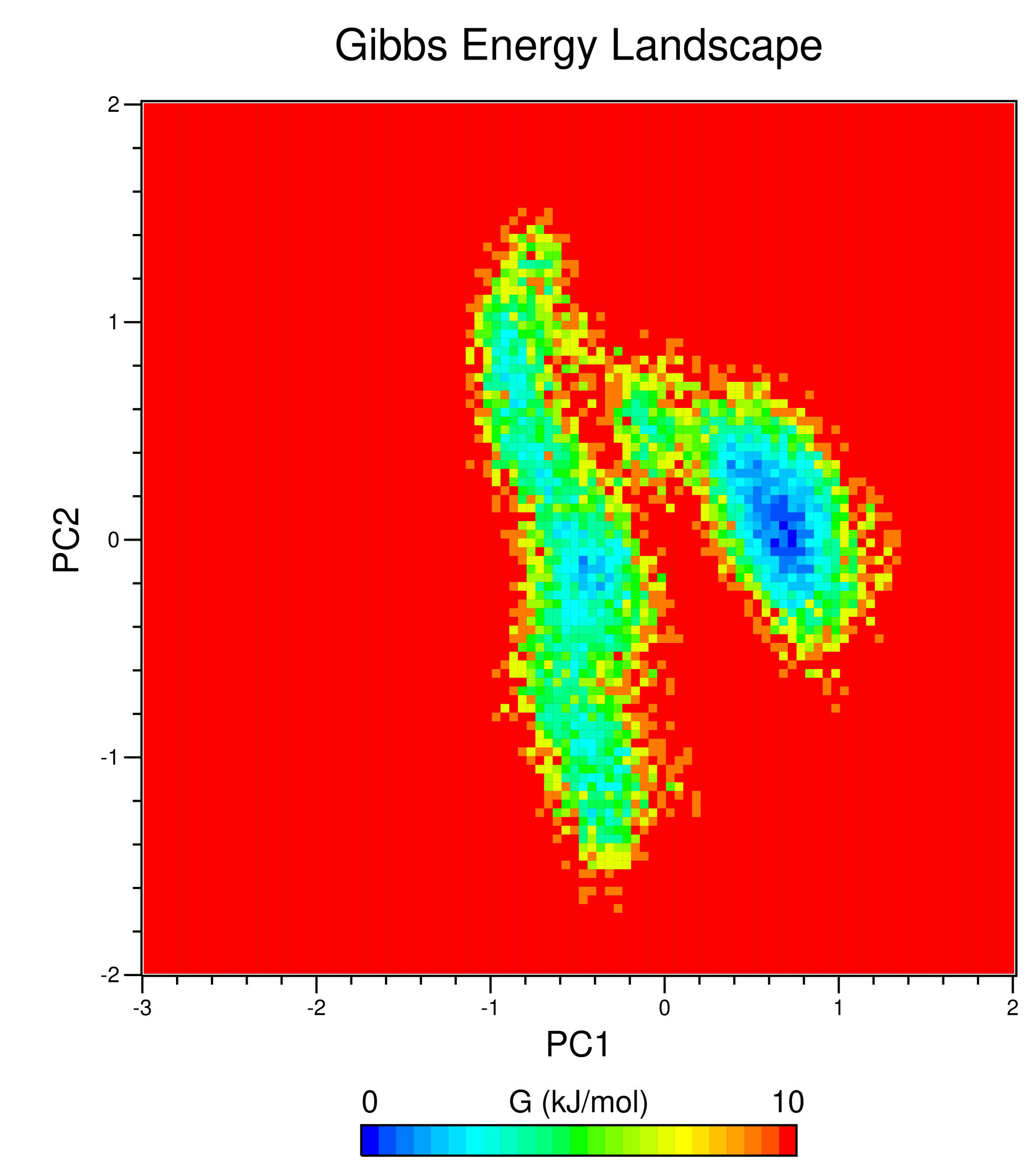

Supplement: S1 File — (ZIP) [file pone.0289046.s003.zip › Supplementary/Compound 8/MD/FEL/Gibbs energy.png]

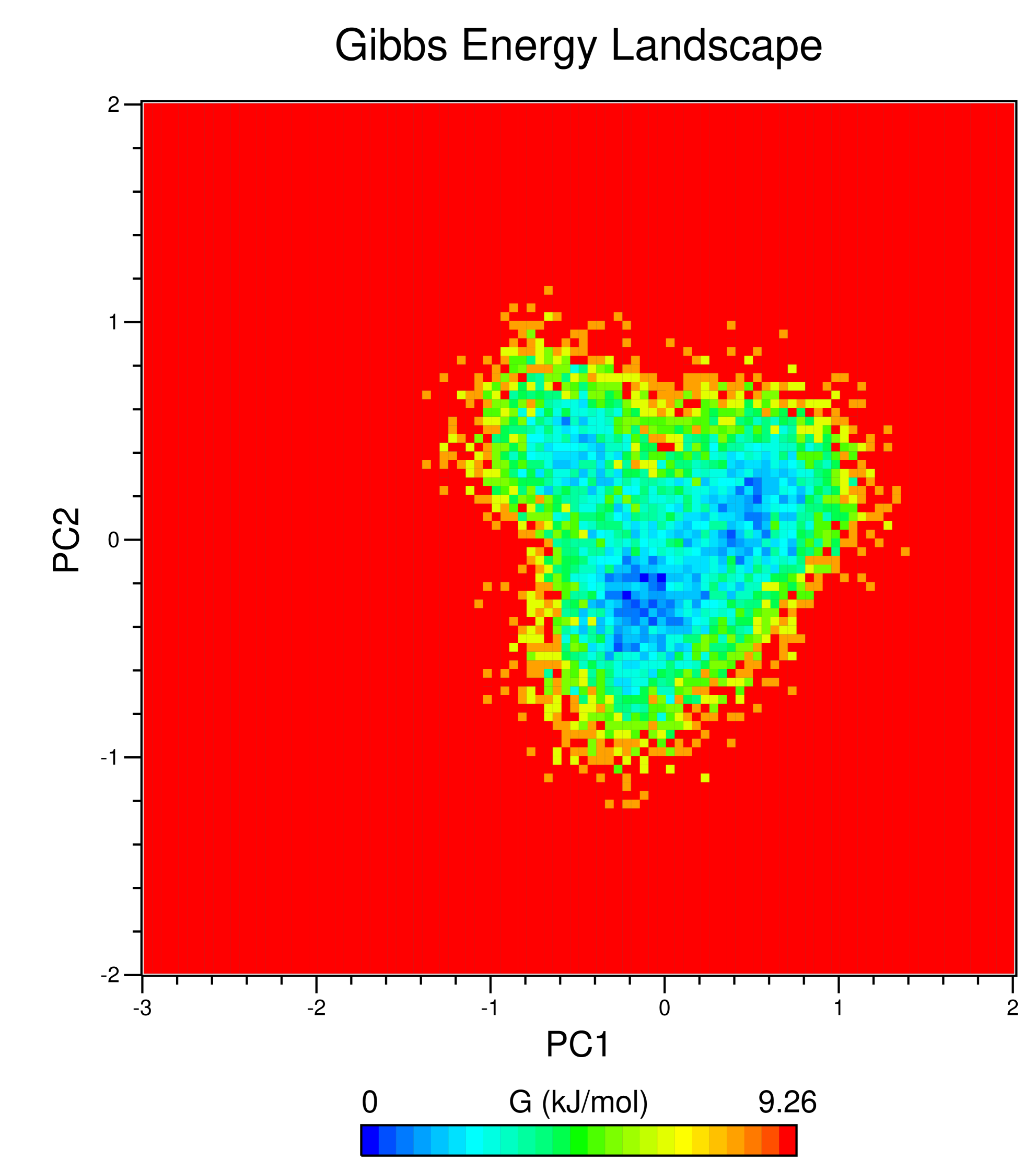

Supplement: S1 File — (ZIP) [file pone.0289046.s003.zip › Supplementary/Free Protein/MD/FEL/gibbs-protein.png]

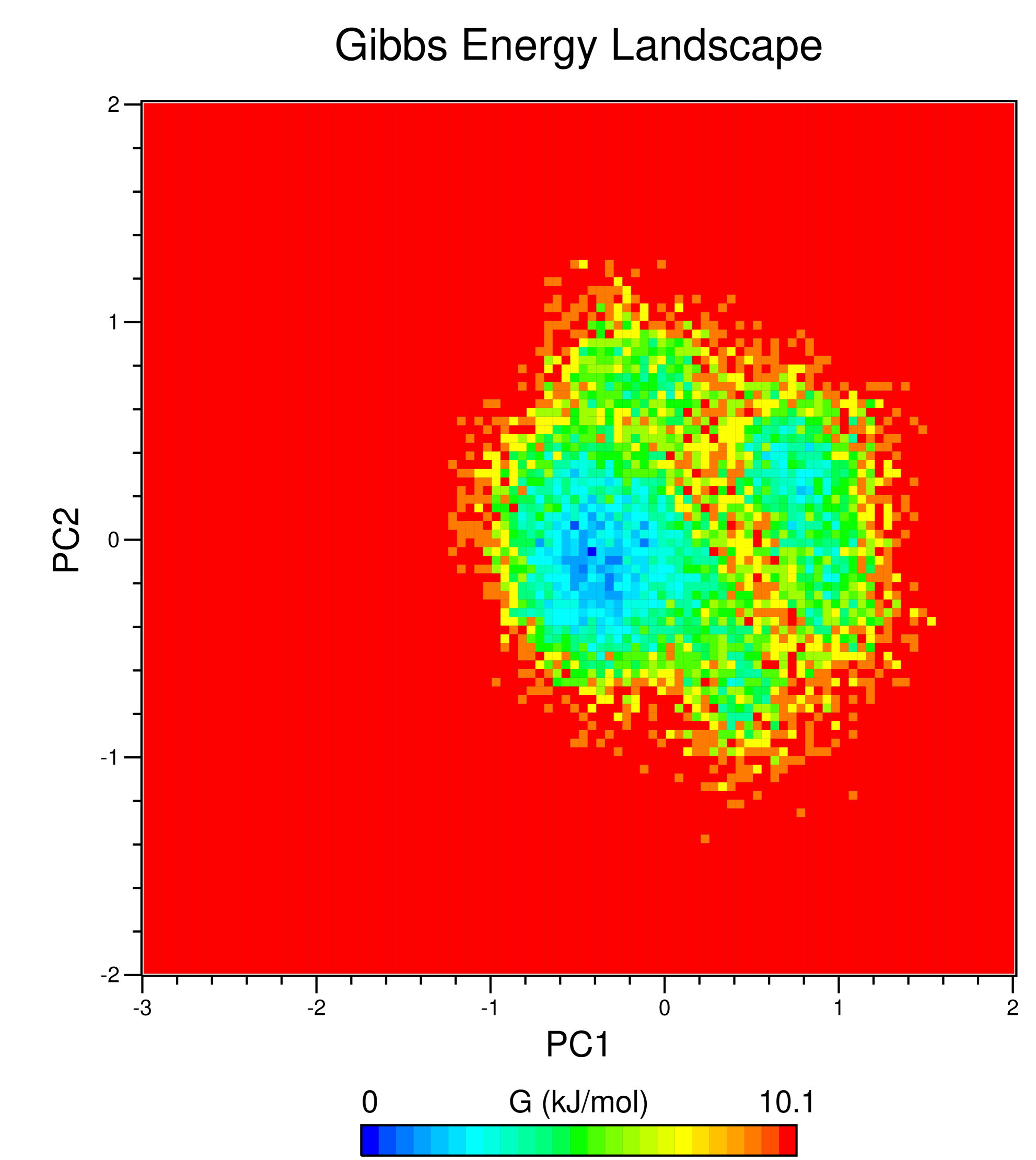

Supplement: S1 File — (ZIP) [file pone.0289046.s003.zip › Supplementary/RAR568/MD/FEL/Gibbs energy.png]
