# Supplementary material for: Discovery of novel RARα agonists using pharmacophore-based virtual screening, molecular docking, and molecular dynamics simulation studies
Source: PLoS One. 2023 Aug 24;18(8):e0289046. doi: 10.1371/journal.pone.0289046 (PMC10449137; doi:10.1371/journal.pone.0289046)
Supplement: S1 File — (ZIP) [file pone.0289046.s003.zip › Supporting information/S1_Table.docx]

**S1 Table. ADMET prediction of 18 selected small molecules.**

**Com. No**. **GI absorption BBB permeant Pgp substrate CYP2D6 & CYP3A4 inhibitor AMES toxicity Hepatotoxicity Skin Sensitization**

| **1 Yes Yes No No No No No**    **2 Yes Yes No No No No No**  **3 Yes Yes No No No No No**  **4 Yes Yes No No No No No**  **5 Yes Yes No No No No No**  **6 Yes Yes No No No No No**    **7 Yes Yes No No No No No**  **8 Yes Yes No No No No No**  **9 Yes Yes No No No No No** |
| --- |

| **10**  **Yes Yes No No No No No**  **11 Yes Yes No No No No No**  **12**  **Yes Yes No No No No No**  **13**  **Yes Yes No No No No No**  **14**  **Yes Yes No No No No No**  **15 Yes Yes No No No No No**  **16 Yes Yes No No No No No**  **17**  **Yes Yes No No No No No**  **18 Yes Yes No No No No No** |
| --- |

**Com. No. GI absorption BBB permeant Pgp substrate CYP2D6 & CYP3A4 inhibitor AMES toxicity Hepatotoxicity Skin Sensitization**
