# Supplementary material for: Discovery of novel RARα agonists using pharmacophore-based virtual screening, molecular docking, and molecular dynamics simulation studies
Source: PLoS One. 2023 Aug 24;18(8):e0289046. doi: 10.1371/journal.pone.0289046 (PMC10449137; doi:10.1371/journal.pone.0289046)
Supplement: S1 File — (ZIP) [file pone.0289046.s003.zip › Supporting information/S2_Table.docx]

**S2 Table. Docking results of RAR568 and 18 selected small molecules in interaction with RARα.**

**Com. No. PubChem ID Docking score H-bond Interaction residues 2D structure**

**(Kcal mol^-1^)**

| ****  **RAR568** [**67042254**](https://pubchem.ncbi.nlm.nih.gov/compound/67042254) **-10.60 3 Ser 232, Ser 287, Arg 276**  ****  **1** [**7464983**](https://pubchem.ncbi.nlm.nih.gov/compound/7464983) **-10.99 3 Ser 232, Ser 287, Arg 276**    ****  **2** [**25219797**](https://pubchem.ncbi.nlm.nih.gov/compound/25219797) **-10.73 3 Ser 232, Ser 287, Arg 276**    ****  **3** [**67977746**](https://pubchem.ncbi.nlm.nih.gov/compound/67977746) **-10.71 2 Ser 287, Arg 276**  ****  **4** [**1713428**](https://pubchem.ncbi.nlm.nih.gov/compound/1713428) **-10.56 3 Ser 232, Ser 287, Arg 276** |
| --- |

**Com. No. PubChem ID Docking score H-bond Interaction residues 2D structure**

**(Kcal mol^-1^)**

|   **5** [**3253473**](https://pubchem.ncbi.nlm.nih.gov/compound/3253473) **-10.17 2 Ser 287, Arg 276**  ****  **6** [**6465258**](https://pubchem.ncbi.nlm.nih.gov/compound/6465258) **-9.64 2 Ser 287, Arg 276**  ****  **7** [**729638**](https://pubchem.ncbi.nlm.nih.gov/compound/729638) **-9.58 2 Ser 287, Arg 276**  ****  **8** [**122024100**](https://pubchem.ncbi.nlm.nih.gov/compound/122024100) **-9.44 3 Ser 232, Ser 287, Arg 276**  ****  **9 91659586 -9.44 2 Ser 287, Arg 272** |
| --- |

**Com. No. PubChem ID Docking score H-bond Interaction residues 2D structure**

**(Kcal mol^-1^)**

| ****  **10** [**121863722**](https://pubchem.ncbi.nlm.nih.gov/compound/121863722) **-9.37 3 Ser 287, Arg 276, Cys 235**  ****  **11** [**121907384**](https://pubchem.ncbi.nlm.nih.gov/compound/121907384) **-9.01 3 Ser 232, Ser 287, Arg 276**  ****  **12 45426604 -8.68 3 Ser 287, Arg 276**  **** |
| --- |

**13** [**14186477**](https://pubchem.ncbi.nlm.nih.gov/compound/14186477) **-8.18 3 Arg 272, Arg 276, Cys 235**

**

**

**14** [**120524598**](https://pubchem.ncbi.nlm.nih.gov/compound/120524598) **-8.04 3 Ser 232, Ser 287, Leu 269**

**Com. No. PubChem ID Docking score H-bond Interaction residues 2D structure**

**(Kcal mol^-1^)**

| ****  **15**  [**11508622**](https://pubchem.ncbi.nlm.nih.gov/compound/11508622) **-7.93 3 Ser 232, Leu 269, Cys 235**  ****  **16**  [**120185142**](https://pubchem.ncbi.nlm.nih.gov/compound/120185142) **-7.73 3 Ser 232, Ser 287, Leu 231**    **17** [**44225907**](https://pubchem.ncbi.nlm.nih.gov/compound/44225907) **-7.27 3 Ser 232, Ser 287, Leu 269**  **** |
| --- |

**18** [**14186458**](https://pubchem.ncbi.nlm.nih.gov/compound/14186458) **-6.79 3 Ser 232, Phe 228, Leu 269**
