# Supplementary material for: Discovery of novel RARα agonists using pharmacophore-based virtual screening, molecular docking, and molecular dynamics simulation studies
Source: PLoS One. 2023 Aug 24;18(8):e0289046. doi: 10.1371/journal.pone.0289046 (PMC10449137; doi:10.1371/journal.pone.0289046)
Supplement: S1 File — (ZIP) [file pone.0289046.s003.zip › Supporting information/S3_Table.docx]

**S3 Table. Average potential energy (kcal.mol^-1^) of RAR568 and the other five selected compounds in interaction with RARα along with free RARα.**

|  | Potential Energy |
| --- | --- |
| RARα | -84128.3777 |
| RAR568 | -85477.0033 |
| Compound 1 | -85421.7047 |
| Compound 2 | -85415.2344 |
| Compound 4 | -85371.8594 |
| Compound 8 | -85411.1244 |
| Compound 11 | -85420.2200 |
