# Supplementary material for: Discovery of novel RARα agonists using pharmacophore-based virtual screening, molecular docking, and molecular dynamics simulation studies
Source: PLoS One. 2023 Aug 24;18(8):e0289046. doi: 10.1371/journal.pone.0289046 (PMC10449137; doi:10.1371/journal.pone.0289046)
Supplement: S1 File — (ZIP) [file pone.0289046.s003.zip › Supporting information/S4_Table.docx]

| **­­ RAR568 ∆G_vdw_ ∆G_elec_ ∆G_solv-polar_ ∆G_solv-nonpol_ ∆G_MMPBSA_** |
| --- |

**S4 Table. Free energies binding of RARα in complex with RAR568 and the other five selected compounds with 10ns interval during 100 ns using MM-PBSA approach.**

**10ns -57.1917 -42.3092 41.3367 -41.0485 -32.1385**

**20ns -55.0126 -55.3942 50.4396 -40.7727 -33.6201**

**30ns -57.2135 -46.9975 46.9532 -40.9933 -31.4170**

**40ns -57.8621 -50.6798 46.8723 -41.0154 -36.0180**

**50ns -56.7838 -46.3129 44.6042 -41.0819 -33.0533**

**60ns -57.3058 -46.6673 45.9966 -40.9680 -32.3613**

**70ns -56.6357 -53.2048 49.5871 -41.2829 -34.6486**

**80ns -57.8207 -53.8518 48.7384 -41.2816 -37.3124**

**90ns -56.7352 -57.7982 52.2371 -40.9252 -35.6942**

**100ns -57.4183 -64.1259 56.2480 -41.1876 -39.4155**

**Compound 1 ∆G_vdw_ ∆G_elec_ ∆G_solv-polar_ ∆G_solv-nonpol_ ∆G_MMPBSA_**

|  |
| --- |

**10ns -48.5227 -51.5201 38.8894 -34.9254 -36.6948**

**20ns -45.2917 -57.1234 36.9081 -34.4866 -38.6050**

**30ns -46.0233 -58.2895 37.0970 -34.4241 -41.6199**

**40ns -46.2745 -61.5555 34.5020 -34.4560 -43.2963**

**50ns -42.9728 -56.1012 34.0176 -33.7266 -44.3353**

**60ns -45.2892 -59.7045 39.4235 -34.4154 -46.8716**

**70ns -43.9470 -53.4663 37.5097 -33.9244 -46.3967**

**80ns -45.8795 -50.5255 34.2011 -34.1299 -47.5317**

**90ns -44.9043 -59.8799 31.9862 -34.3160 -47.1957**

**100ns -46.8888 -55.1673 31.6839 -34.3897 -45.4115**

**Compound 2 ∆G_vdw_ ∆G_elec_ ∆G_solv-polar_ ∆G_solv-nonpol_ ∆G_MMPBSA_**

|  |
| --- |

**10ns -45.2238 -49.9151 42.8906 -33.3545 -31.8079**

**20ns -45.9333 -53.9603 46.9248 -33.4552 -32.1639**

**30ns -46.3022 -57.9341 42.7691 -33.4763 -40.5104**

**40ns -45.2644 -62.4026 47.1390 -33.3165 -40.5127**

**50ns -45.6611 -57.2832 42.1503 -33.2530 -40.1621**

**60ns -46.0185 -53.9187 43.1292 -33.3159 -36.7560**

**70ns -45.4780 -53.1483 43.2495 -33.2214 -35.1647**

**80ns -44.4880 -56.9872 46.2782 -33.1719 -35.1150**

**90ns -44.2922 -54.6884 43.2588 -33.1150 -36.3446**

**100ns -44.8772 -59.8801 50.0551 -33.0395 -34.2577**

**Compound 4 ∆G_vdw_ ∆G_elec_ ∆G_solv-polar_ ∆G_solv-nonpol_ ∆G_MMPBSA_**

|  |
| --- |

**10ns -43.9238 -39.0453 33.0918 -31.8169 -27.9256**

**20ns -45.0803 -41.1784 37.2641 -31.9270 -27.2310**

**30ns -44.7312 -33.3689 29.3645 -31.8139 -26.5479**

**40ns -45.0664 -28.3036 27.3803 -31.7036 -23.4680**

**50ns -43.4540 -37.5042 32.8036 -31.6802 -25.7303**

**60ns -43.4543 -34.9266 28.5890 -31.3897 -27.5669**

**70ns -43.9442 -39.0503 31.4267 -31.5249 -29.1315**

**80ns -43.5066 -42.1739 38.2713 -31.4465 -35.7917**

**90ns -42.4097 -46.1984 40.0838 -31.5042 -36.2802**

**100ns -42.3553 -42.5347 39.2140 -31.5719 -33.7063**

**Compound 8 ∆G_vdw_ ∆G_elec_ ∆G_solv-polar_ ∆G_solv-nonpol_ ∆G_MMPBSA_**

|  |
| --- |

**10ns -43.1642 -41.0258 35.7457 -30.4356 -26.0276**

**20ns -43.1108 -44.9601 37.1715 -30.5609 -28.5994**

**30ns -42.5124 -46.0664 36.2310 -30.5212 -29.7060**

**40ns -43.4867 -41.5195 36.7508 -30.4867 -25.7447**

**50ns -44.1662 -40.1621 35.3726 -30.9293 -26.8177**

**60ns -44.5332 -35.8946 30.1130 -30.9110 -28.2973**

**70ns -45.0343 -43.3751 37.2788 -30.7939 -28.9928**

**80ns -45.1525 -49.5019 39.4981 -30.9292 -32.6365**

**90ns -45.0211 -47.8989 36.4723 -30.8634 -33.8610**

**100ns -44.2363 -48.1385 40.6000 -30.8488 -29.5270**

**Compound 11 ∆G_vdw_ ∆G_elec_ ∆G_solv-polar_ ∆G_solv-nonpol_ ∆G_MMPBSA_**

|  |
| --- |

**10ns -41.4085 -50.7739 42.6234 -29.5925 -27.3354**

**20ns -40.9568 -53.4442 37.4904 -29.4602 -34.6998**

**30ns -41.9013 -61.7611 51.3275 -29.6870 -29.9907**

**40ns -41.7309 -55.0653 45.3125 -29.7819 -29.5862**

**50ns -41.5470 -53.0536 45.1743 -29.9869 -28.1786**

**60ns -39.2004 -57.8092 45.4377 -29.5956 -31.4275**

**70ns -40.9677 -54.4045 44.9418 -29.2497 -28.9232**

**80ns -41.0863 -51.9889 42.0589 -29.5810 -28.9480**

**90ns -41.4742 -53.1215 43.0989 -29.5407 -29.4142**

**100ns -41.6243 -55.7559 47.1852 -29.6046 -28.4018**
