# Supplementary material for: Discovery of novel RARα agonists using pharmacophore-based virtual screening, molecular docking, and molecular dynamics simulation studies
Source: PLoS One. 2023 Aug 24;18(8):e0289046. doi: 10.1371/journal.pone.0289046 (PMC10449137; doi:10.1371/journal.pone.0289046)
Supplement: S1 File — (ZIP) [file pone.0289046.s003.zip › Response to Reviewers.docx]

Dear Dr. Nafees Ahemad,

Thank you for giving us the opportunity to submit the revised version of the manuscript entitled **“Discovery of Novel Therapeutic RARα Agonists Through Ligand-Based Pharmacophore Modeling, Virtual Screening, Molecular Docking, and MD Simulation Studies”.**

We appreciate the time and effort that you and the reviewers have dedicated to provide valuable feedbacks on our manuscript. We obliged ourselves to respond all the suggestions provided by you and the reviewers to increase the quality and clarity of the manuscript. All the changes according to the reviewers’ comments are highlighted in yellow throughout the manuscript. The whole manuscript was also revised grammatically.

We also have a request which may not be very unusual for the academics and researchers in Iran. The tough economic conditions in the last decade, has made it impossible for us to publish good works in high ranking journals. Sadly, the publication fees are usually equal to an average professor salary in Iran, thus, practically unaffordable. If it is possible for this journal, we will really be grateful to be exempted from publication fee payment.

We look forward to hearing from you soon regarding our submission and to respond to any further questions and comments you may have. In continue there is a point-by-point response to the reviewers’ comments and concerns.

Sincerely,

Afshin Fassihi

PharmD. PhD

------------------------------ Original Message ------------------------------
from: PLOS <[em@editorialmanager.com](mailto:em@editorialmanager.com)>
to: Afshin <[fassihi@pharm.mui.ac.ir](mailto:fassihi@pharm.mui.ac.ir)>
date: Wednesday، 18 Aabaan 1401 1:19:23
title: PLOS ONE Decision: Revision required [PONE-D-22-25119] - [EMID:a85affd6da757323]

PONE-D-22-25119

Discovery of Novel Therapeutic RARα Agonists Through Ligand-Based Pharmacophore Modeling, Virtual Screening, Molecular Docking, and MD Simulation Studies

PLOS ONE

Dear Dr. Fassihi,

Thank you for submitting your manuscript to PLOS ONE. After careful consideration, we feel that it has merit but does not fully meet PLOS ONE’s publication criteria as it currently stands. Therefore, we invite you to submit a revised version of the manuscript that addresses the points raised during the review process.

A significant changes/validation is required in the manuscript.

Please address the reviewer's comments.

Please submit your revised manuscript by Dec 24 2022 11:59PM. If you will need more time than this to complete your revisions, please reply to this message or contact the journal office at  [plosone@plos.org](mailto:plosone@plos.org). When you're ready to submit your revision, log on to <https://www.editorialmanager.com/pone/> and select the 'Submissions Needing Revision' folder to locate your manuscript file.

Please include the following items when submitting your revised manuscript:

- A rebuttal letter that responds to each point raised by the academic editor and reviewer(s). You should upload this letter as a separate file labeled 'Response to Reviewers'.
- A marked-up copy of your manuscript that highlights changes made to the original version. You should upload this as a separate file labeled 'Revised Manuscript with Track Changes'.
- An unmarked version of your revised paper without tracked changes. You should upload this as a separate file labeled 'Manuscript'.

If you would like to make changes to your financial disclosure, please include your updated statement in your cover letter. Guidelines for resubmitting your figure files are available below the reviewer comments at the end of this letter.

If applicable, we recommend that you deposit your laboratory protocols in [protocols.io](http://protocols.io/) to enhance the reproducibility of your results. Protocols.io assigns your protocol its own identifier (DOI) so that it can be cited independently in the future. For instructions see: <https://journals.plos.org/plosone/s/submission-guidelines#loc-laboratory-protocols>. Additionally, PLOS ONE offers an option for publishing peer-reviewed Lab Protocol articles, which describe protocols hosted on [protocols.io](http://protocols.io/). Read more information on sharing protocols at <https://plos.org/protocols?utm_medium=editorial-email&utm_source=authorletters&utm_campaign=protocols>.

We look forward to receiving your revised manuscript.

Kind regards,

Nafees Ahemad
Academic Editor
PLOS ONE

Journal Requirements:

When submitting your revision, we need you to address these additional requirements.

1. Please ensure that your manuscript meets PLOS ONE's style requirements, including those for file naming. The PLOS ONE style templates can be found at

<https://journals.plos.org/plosone/s/file?id=wjVg/PLOSOne_formatting_sample_main_body.pdf> and

<https://journals.plos.org/plosone/s/file?id=ba62/PLOSOne_formatting_sample_title_authors_affiliations.pdf>

**Response:** We studied all the PLOS ONE's style requirements and set the manuscript and other files in that format.

2. We noticed you have some minor occurrence of overlapping text with the following previous publication(s), which needs to be addressed:

<https://www.sciencedirect.com/science/article/abs/pii/S0960894X16305418?via%3Dihub>

<https://kclpure.kcl.ac.uk/ws/files/83192231/Design_and_synthesis_of_CLARKE_Publishedonline9December2017_GREEN_AAM_CC_BY_NC_ND_.pdf>

<https://journals.plos.org/plosone/article?id=10.1371%2Fjournal.pone.0217031>

**Response:** The text that had been added to the manuscript from the mentioned publications were paraphrased, and the corresponding references were added to the Reference part of the manuscript.

In your revision ensure you cite all your sources (including your own works), and quote or rephrase any duplicated text outside the methods section. Further consideration is dependent on these concerns being addressed.

3. In your Data Availability statement, you have not specified where the minimal data set underlying the results described in your manuscript can be found. PLOS defines a study's minimal data set as the underlying data used to reach the conclusions drawn in the manuscript and any additional data required to replicate the reported study findings in their entirety. All PLOS journals require that the minimal data set be made fully available. For more information about our data policy, please see <http://journals.plos.org/plosone/s/data-availability>.

Upon re-submitting your revised manuscript, please upload your study’s minimal underlying data set as either Supporting Information files or to a stable, public repository and include the relevant URLs, DOIs, or accession numbers within your revised cover letter. For a list of acceptable repositories, please see <http://journals.plos.org/plosone/s/data-availability#loc-recommended-repositories>. Any potentially identifying patient information must be fully anonymized.

**Response:** All the minimal data set underlying the results described in our manuscript will be uploaded as Supporting Information files.

Important: If there are ethical or legal restrictions to sharing your data publicly, please explain these restrictions in detail. Please see our guidelines for more information on what we consider unacceptable restrictions to publicly sharing data: <http://journals.plos.org/plosone/s/data-availability#loc-unacceptable-data-access-restrictions>. Note that it is not acceptable for the authors to be the sole named individuals responsible for ensuring data access.

We will update your Data Availability statement to reflect the information you provide in your cover letter.

**Response: Data Availability:** All relevant data are within the manuscript and its [Supporting information](https://journals.plos.org/plosone/article?id=10.1371/journal.pone.0275432#sec021) files.

4. PLOS requires an ORCID iD for the corresponding author in Editorial Manager on papers submitted after December 6th, 2016. Please ensure that you have an ORCID iD and that it is validated in Editorial Manager. To do this, go to ‘Update my Information’ (in the upper left-hand corner of the main menu), and click on the Fetch/Validate link next to the ORCID field. This will take you to the ORCID site and allow you to create a new iD or authenticate a pre-existing iD in Editorial Manager. Please see the following video for instructions on linking an ORCID iD to your Editorial Manager account: <https://www.youtube.com/watch?v=_xcclfuvtxQ>

**Response:** The ORCID IDs for the corresponding authors were provided in the submission process.

5. Please ensure that you refer to Figures 5, 6, 7, 8 and 11 in your text as, if accepted, production will need this reference to link the reader to the figure.

**Response:** These Figures are now referred in the manuscript.

[Note: HTML markup is below. Please do not edit.]

Reviewers' comments:

Reviewer's Responses to Questions

**Comments to the Author**

1. Is the manuscript technically sound, and do the data support the conclusions?

The manuscript must describe a technically sound piece of scientific research with data that supports the conclusions. Experiments must have been conducted rigorously, with appropriate controls, replication, and sample sizes. The conclusions must be drawn appropriately based on the data presented.

Reviewer #1: Yes

Reviewer #2: Partly

2. Has the statistical analysis been performed appropriately and rigorously?

Reviewer #1: Yes

Reviewer #2: Yes

3. Have the authors made all data underlying the findings in their manuscript fully available?

The [PLOS Data policy](http://www.plosone.org/static/policies.action#sharing) requires authors to make all data underlying the findings described in their manuscript fully available without restriction, with rare exception (please refer to the Data Availability Statement in the manuscript PDF file). The data should be provided as part of the manuscript or its supporting information, or deposited to a public repository. For example, in addition to summary statistics, the data points behind means, medians and variance measures should be available. If there are restrictions on publicly sharing data—e.g. participant privacy or use of data from a third party—those must be specified.

Reviewer #1: No

Reviewer #2: Yes

4. Is the manuscript presented in an intelligible fashion and written in standard English?

PLOS ONE does not copyedit accepted manuscripts, so the language in submitted articles must be clear, correct, and unambiguous. Any typographical or grammatical errors should be corrected at revision, so please note any specific errors here.

Reviewer #1: Yes

Reviewer #2: No

5. Review Comments to the Author

Please use the space provided to explain your answers to the questions above. You may also include additional comments for the author, including concerns about dual publication, research ethics, or publication ethics. (Please upload your review as an attachment if it exceeds 20,000 characters)

Reviewer #1: General Comments:

The structure of the introduction resembles thesis or dissertation format. Please kindly refer to the author guideline for a proper journal format, or you may benchmark to PLOSone existing publications.

**Response:** We would like to thank the Reviewer for his/her comments. We changed the format of the Introduction in a way that matches to the journal style.

There should be no figure at all in the intro section

**Response:** All the figures are now eliminated from the introduction section.

The attached figures are in poor quality. Please kindly improve the resolution accordingly!

**Response:** The quality of figures are now improved.

Specific comments:

How did you validate your docking methods? You have to validate the docking method if you want to proceed with this protocol. There are two conditions that you have to take care of:
a. Provided that there is a naturally attached ligands in the protein. You should redock the ligand, and compare the binding energy with your leads.
b. Provided that there is no attached ligands in the protein, as in the example of the homology modeling result. You have to use DUDE database decoy to validate your docking protocol. As a lead, you may use a compound that has been proven in the wet lab for targeting the enzyme. Please check this link: <http://dude.docking.org/>

**Response:** The pdb crystallographic structures of the proteins were in complex with their own selective ligands. We redocked the selective ligand for each protein and observed the final RMSD. The RMSDs were under 2 and acceptable.

Write down PUBCHEM ID or CHEMSPIDER ID of the compounds so readers could easily navigate their properties in the online database. Revise the table to include the IDs.

**Response:** The related IDs of the compounds are now added to the docking table.

Reviewer #2: Manuscript Title: Discovery of Novel Therapeutic RARα Agonists through Ligand-Based Pharmacophore Modeling, Virtual Screening, Molecular Docking, and MD Simulation Studies
Reviewer Recommendation: Major revision required.

Major comments

1. Primary check on this manuscript for language refinement is mandatory as I see that the English is not satisfactory, and moreover, there are more typographical errors. I suggest the authors go with language and grammar checks.

**Response:** The whole manuscript has been revised grammatically to eliminate the typographical and grammatical errors.

2. Authors requested to concise the introduction part with key points which are more relevant to the title of the manuscript.

**Response:** The Introduction has been changed to be more brief and comprehensive.

3. In the methods section authors have mentioned that both SBDD and LBDD were carried out, but the detailed methodology was lacking.

**Response:** In this research project molecular docking and dynamic simulations are categorized as Structure-Based methods. The pharmacophore search in this study was carried out in Pharmit server. This server performs pharmacophore search based on the model made by the most important pharmacophores of the lead compound’s structure in complex with the protein. Thus, it can be categorized as a ligand-based method. We selected pharmacophore search option in Pharmit to search the provided databases in this platform and find the matching compounds. The pharmacophores are defined in this web server based on the ligand-protein interactions. Pharmacophores are created and edited in a modern interactive interface powered by 3Dmol.js in this web server.

4. Provide the detailed information on the number of compounds or ligand sources that are used for prediction pharmacophore modeling in ligand based drug design.

**Response:** For all the RAR receptors there are crystallographic structures in complex with their selective agonists. After redocking of the selective agonists and validation of the docking process, the docking pose with the best energy of the protein-ligand complex was selected for the pharmacophore search query in the Pharmit server. This server makes a pharmacophore model based on the essential features of an interaction of the ligand with the protein. This model will be the base of pharmacophore search in the available databases using the Pharmer. A pharmacophore query specifies the features using tolerance spheres. Compounds match if they can be positioned so that their corresponding features are located within these spheres. After generation of the pharmacophore features some of them can be eliminated by the user’s choice. Results from screening of the databases are aligned to the pharmacophore to minimize the root mean squared deviation (RMSD) between the query features and the hit compound features. Results are sorted with respect to this pharmacophore RMSD. Thus, in this method, several molecules are not used to make a pharmacophore model.

5. Authors have mentioned that structures of all 18 selected small molecules were drawn using ChemDraw software, providing detailed information on how authors have selected these 18 molecules and their structural information.

**Response:** Virtual screening is a lead discovery technique that plays an important role in the discovery of potential compounds. By this method a large number of compounds can be screened virtually considering the residues present in the active site of a target. It enables the screening of large data sets to find the structures possessing the highest tendency to bind to the target. AutoDock vina is a molecular docking tool which determines the preferred relative orientation of ligand during its docking on target protein, and form a stable complex with lowest binding energy. In this study, 938 compounds obtained from ADMET in silico evaluation were screened against the RARα target, and compounds with the best favorable binding energies were selected. The top 18 compounds were selected based on lowest binding energy which ranged between −11.7 Kcal/mol and −10.7 Kcal/mol, considering the presence of all the critical chemical interactions with the RARα protein. .

6. The study used the 10 different databases, please use the references indicated by the developers.

**Response:** Pharmit has pre-built libraries generated from CHEMBL21, ChemDiv (www.chemdiv.com), Mol-Port (www.molport.com), the NCI Open Chemical Repository (dtp.cancer.gov), PubChem, and so on. The list of these databases is added in the manuscript with their corresponding references.

7. It is possible to obtain the 10 different databases through the pre-established subsets or tranches. Specify how to obtain the compounds from the 10 different and the number of compounds used in this study.

**Response:** After the pharmacophore generation by the Pharmit server based on the essential features of ligand-protein interactions and specifying the features using tolerance spheres, the data bases are screened using the Pharmer. Compounds match if they can be positioned so that their corresponding features are located within these spheres. Results from screening of the databases are aligned to the pharmacophore to minimize the root mean squared deviation (RMSD) between the query features and the hit compound features. More filtrations, such as molecular weight, the number of rotatable bonds, log P, topological polar surface area, the number of aromatic groups, the number of hydrogen bond acceptors, and the number of hydrogen bond donors are applied. Properties are precomputed using Open Babel. After this process, the final compounds are saved for other steps of the study. The result of pharmacophore search was a collection containing 12975 linear small molecules with MWT ≤ 500 g/mol, LogP ≤ 5, RMSD ≤ 2, and Energy Minimization -9≥, which were considered for the ADMET prediction steps.

8. In ligand preparation, how they got the compounds, if they draw, cite it properly along with how they positioned those drawn compounds focus on the XYZ axis (As the 18 compounds must be positioned exactly at a single point to match pharmacophore).

**Response:** As explained in the previous responses, the pharmacophore model was prepared according to the essential features of the lead ligand-protein interactions by the Pharmit webserver. After that, this model was used for the screening process in the available databases in the pharmit server. The best results in this step were used for the ADMET prediction. Then the following investigations were carried out by the PyRx software. Eighteen compounds with the best energies were selected for the docking with Autodock to investigate the interactions in detail. These 18 compounds were drawn by ChemDraw, energy minimized in HyperChem8 program (MM+ force field and PM3 semiempirical calculations), and used for molecular docking and then MD calculations.

9. Provide detailed information on maximum and minimum pharmacophore hypothesis were set to predict a good model and how authors have selected the best pharmacophore hypothesis and features were selected for further study.

**Response:** The pharmacophore model was generated by the Pharmit server based on the essential features of the lead ligand-protein interactions and specifying the features using tolerance spheres. The compounds in the online databases which located within the pharmacophore spheres were the output of the screening process (we added some other filters with this screening which was explained in the manuscript). Thus, we did not select the pharmacophore hypothesis, but we could select the essential pharmacophore features, which are listed in the Results & discussion part of the manuscript and Fig. 1.

10. Did authors have done any pharmacophore validation?

**Response:** According to the response to the previous questions, the pharmacophore model was built by the Pharmit server and was used for the virtual screening in online databases. This web server is very trustworthy. The article introducing this webserver was published in the prestigious journal of “nucleic acids research” with impact factor of 19.160. Relying on this article (doi: 10.1093/nar/gkw287), we trusted the pharmacophore model and used it for the pharmacophore virtual screening.

11. Authors requested to provide information on chemistry and mode of action of best ligands and their interaction analysis in more detail.

**Response:** More explanations about the mode of action of best ligands and their analysis were added to the manuscript.

12. Did authors have noticed any structural similarity and all the top ranked compounds share the similar binding pocket of RARα?

**Response:** Based on a pharmacophore search of the pharmacophore features and molecular shape of RAR568, all obtained compounds shared similar structural features. All of them contain one carboxylate moiety as well as one carbonyl moiety to present HBDs, one amide moiety to represent HBA, and two aromatic rings to represent hydrophobic centroids, which are located in the same spacial position. Moreover, the key interactions of RAR568 with amino acid residues at the binding site of RARα were considered. To this end, top ranked compounds especially compound 1, 2, 4, 8, and 11 shared the similar binding pocket of RARα compare to RARα-RAR568 complex. All of them interact with ARG 276, SER287, and RARα-specific SER 232.

13. In Stable 2 it was noticed that compound 1 to 5 shows more similar docking score and compound 11 shows less score compared to 3 and other compounds, what basis authors have selected 1, 2, 4, 8 and 11 for further study?

**Response:** Compounds 1, 2, 4, 8, and 11 shared the most similar important interactions, and key pharmacophore point matching compared to the reference RARα-RAR568 complex. Analysis of RARα-RAR568 as well as RARα-the five selected compounds indicated that RAR568 and all the selected compounds formed 3 hydrogen bonds with the key residues of the RARα binding site. One hydrogen bond was formed between the nitrogen atom of the amide moiety of RAR568 with RARα-specific SER 232. The same interaction was also formed between this amino acid and the five selected compounds. Some other Hydrogen bonds were also formed between the oxygen atoms of the carboxylate moiety of RAR568 and the five selected compounds with SER 287 and ARG 276 key residues. Therefore, these compounds with the most important interactions with the key amino acid residues of the RAR binding site and the best pharmacophore point matching were selected for further analysis.

14. I suggest authors replace that 2D interaction pattern with 3D figures with good clarity or at least 600 DPI.

**Response:** The 2D interaction patterns were replaced with the 3D structures, and the qualities were improved.

15. More results, but very less discussion is not advisable, I request the author to give a brief discussion of the findings.

**Response:** More disscusion on the results are now added to the manuscript.

16. Figures should be given more clear way

**Response:** The quality of the figures are now improved.

17. MD simulation with 10ns interval MM/PBSA energy for all the complex MD will be good for validating the study

**Response:** The MM/PBSA energy calculations were carried out for all the six complexes with 10ns interval, and the results are now added to the manuscript.

6. PLOS authors have the option to publish the peer review history of their article. If published, this will include your full peer review and any attached files.


If you choose “no”, your identity will remain anonymous but your review may still be made public.

**Do you want your identity to be public for this peer review?** For information about this choice, including consent withdrawal, please see our [Privacy Policy](https://www.plos.org/privacy-policy).

Reviewer #1: No

Reviewer #2: No

[NOTE: If reviewer comments were submitted as an attachment file, they will be attached to this email and accessible via the submission site. Please log into your account, locate the manuscript record, and check for the action link "View Attachments". If this link does not appear, there are no attachment files.]

While revising your submission, please upload your figure files to the Preflight Analysis and Conversion Engine (PACE) digital diagnostic tool, <https://pacev2.apexcovantage.com/>. PACE helps ensure that figures meet PLOS requirements. To use PACE, you must first register as a user. Registration is free. Then, login and navigate to the UPLOAD tab, where you will find detailed instructions on how to use the tool. If you encounter any issues or have any questions when using PACE, please email PLOS at [figures@plos.org](mailto:figures@plos.org). Please note that Supporting Information files do not need this step.
